# p38-TFEB pathways promote microglia activation through inhibiting CMA-mediated NLRP3 degradation in Parkinson's disease

**DOI:** 10.1186/s12974-021-02349-y

**Published:** 2021-12-20

**Authors:** Jialong Chen, Kanmin Mao, Honglin Yu, Yue Wen, Hua She, He Zhang, Linhua Liu, Mingque Li, Wenjun Li, Fei Zou

**Affiliations:** 1 Department of Occupational Health and Occupational Medicine, Guangdong Province Key Laboratory of Tropical Disease Research, School of Public Health, Southern Medical University, No. 1838, North Guangzhou Road, Guangzhou, 510515 Guangdong Province China; 2 School of Public Health, Guangdong Medical University, Dongguan,, Guangdong Province China; 3 Department of Pharmacology, Emory University School of Medicine, Atlanta, GA USA

**Keywords:** Parkinson’s disease, p38, NLRP3, Chaperone-mediated autophagy, TFEB, NLRP3

## Abstract

**Background:**

Parkinson’s disease (PD) is characterized by degeneration of dopaminergic neurons in the substantia nigra pars compacta (SNpc), accompanied by accumulation of α-synuclein, chronic neuroinflammation and autophagy dysfunction. Previous studies suggested that misfolded α-synuclein induces the inflammatory response and autophagy dysfunction in microglial cells. The NLRP3 inflammasome signaling pathway plays a crucial role in the neuroinflammatory process in the central nervous system. However, the relationship between autophagy deficiency and NLRP3 activation induced by α-synuclein accumulation is not well understood.

**Methods:**

Through immunoblotting, immunocytochemistry, immunofluorescence, flow cytometry, ELISA and behavioral tests, we investigated the role of p38-TFEB-NLRP3 signaling pathways on neuroinflammation in the α-synuclein A53T PD models.

**Results:**

Our results showed that increased protein levels of NLRP3, ASC, and caspase-1 in the α-synuclein A53T PD models. P38 is activated by overexpression of α-synuclein A53T mutant, which inhibited the master transcriptional activator of autophagy TFEB. And we found that NLRP3 was degraded by chaperone-mediated autophagy (CMA) in microglial cells. Furthermore, p38-TFEB pathways inhibited CMA-mediated NLRP3 degradation in Parkinson's disease. Inhibition of p38 had a protective effect on Parkinson's disease model via suppressing the activation of NLRP3 inflammasome pathway. Moreover, both p38 inhibitor SB203580 and NLRP3 inhibitor MCC950 not only prevented neurodegeneration in vivo, but also alleviated movement impairment in α-synuclein A53T-tg mice model of Parkinson’s disease.

**Conclusion:**

Our research reveals p38-TFEB pathways promote microglia activation through inhibiting CMA-mediated NLRP3 degradation in Parkinson's disease, which could be a potential therapeutic strategy for PD.

**Graphical abstract:**

p38-TFEB pathways promote microglia activation through inhibiting CMA-mediated NLRP3 degradation in Parkinson's disease. In this model, p38 activates NLRP3 inflammasome via inhibiting TFEB in microglia. TFEB signaling negatively regulates NLRP3 inflammasome through increasing LAMP2A expression, which binds to NLRP3 and promotes its degradation via chaperone-mediated autophagy (CMA). NLRP3-mediated microglial activation promotes the death of dopaminergic neurons.

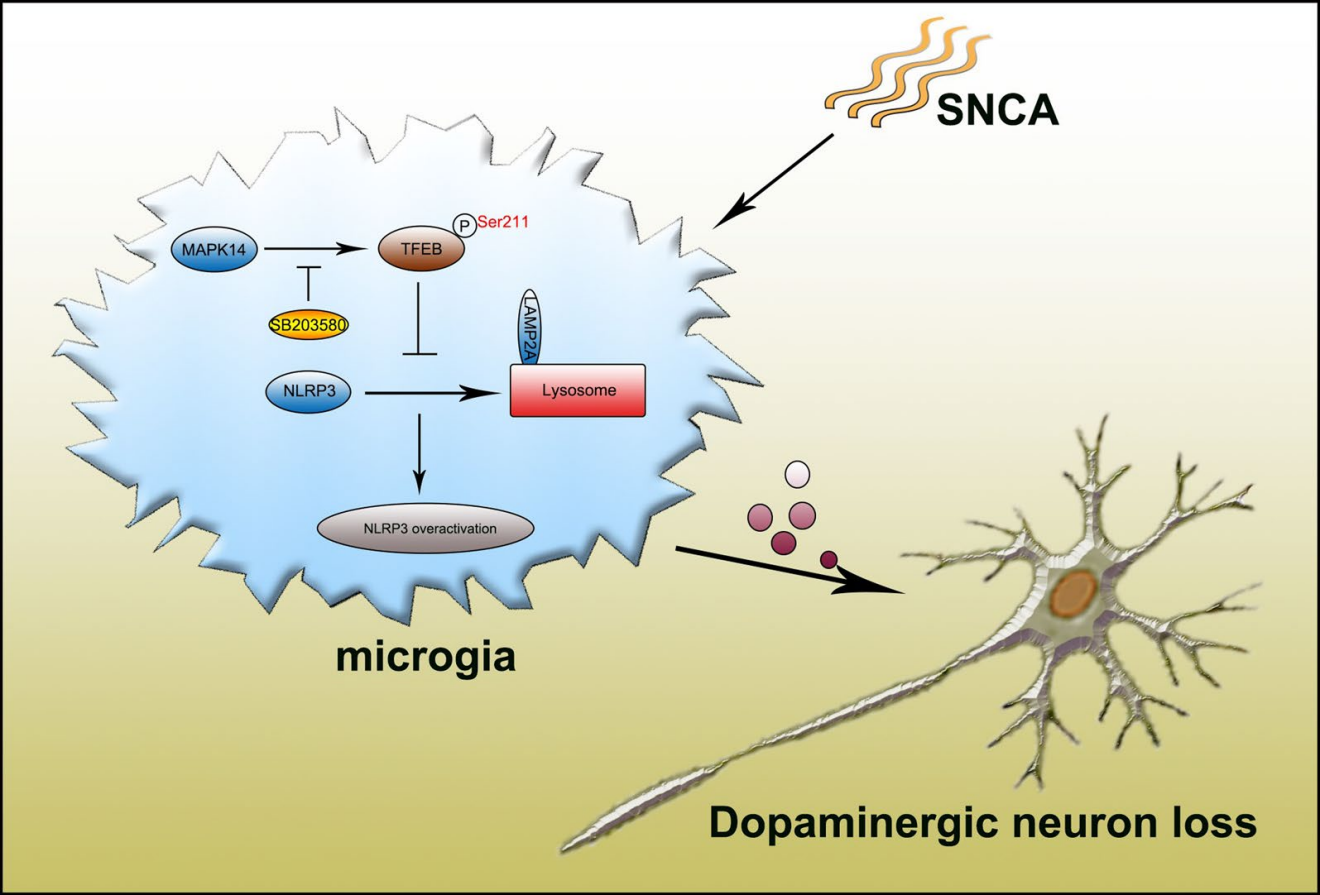

**Supplementary Information:**

The online version contains supplementary material available at 10.1186/s12974-021-02349-y.

## Background

Parkinson’s disease (PD) is characterized by the loss of dopaminergic neurons in the substantia nigra, resulting in neuromuscular dysfunction such as bradykinesia, muscular rigidity and rest tremors. Previous studies have shown that neuroinflammation may be a primary cause of PD-related pathogenesis and the degeneration and loss of dopaminergic neurons. Proinflammatory cytokines such as tumor necrosis factor α (TNF-α) and IL-1β are upregulated in the cerebrospinal fluid and blood of PD patients [1]. Moreover, activated microglia release proinflammatory cytokines that lead to neuronal death, in turn, dopaminergic neurons loss exacerbates neuroinflammation. This vicious cycle is closely associated with the pathological process of PD. The NLRP3 inflammation complex activates the innate immune response and causes cell pyroptosis [2, 3]. Recent studies demonstrated that NLRP3 inflammasome is involved in neurodegenerative diseases and NLRP3 deficiency plays a protective role in animal models of Alzheimer’s disease (AD) and PD [4, 5]. Although the mechanisms of NLRP3 inflammasome in diverse inflammatory diseases have been extensively investigated, its regulatory networks in microglia are unclear.

Mitogen-activated protein kinases (MAPKs) regulate various cellular processes. The p38 mitogen-activated protein kinase (p38MAPK), a prominent member of the MAPKs, is not only involved in cell cycle, cell death, development, differentiation, senescence and tumorigenesis, but also functions as a specific class of serine/threonine kinase regulating the inflammation responses. The important role of p38-mediated inflammatory responses was originally confirmed as the target of the pyridinyl imidazole that inhibited inflammatory cytokines release in lipopolysaccharides (LPS)-treated monocytes [6]. The MAPK pathways contributed to the increased production of proinflammatory cytokines in microglia that were treated with toll-like receptor (TLR) ligands or beta-amyloid (Aβ) [7]. Recently, Mao et al. identified that p38 inhibits autophagy and promotes microglial inflammatory responses via ULK1 phosphorylation [8]. A few studies have demonstrated that pharmacological inhibition of p38 plays a protective role in several animal models of neurodegenerative disease [9–11]. Moreover, studies in vivo have also shown that p38 deficiency protects from aggravating AD progress via enhancing the level of autophagy [12, 13]. The discovery of transcription factor EB (TFEB) has triggered more and more studies that try to explore the therapeutic potential of targeting TFEB to treat neurodegenerative diseases, because it works as a master regulator of autophagy [14]. Although the protective effect of p38 inhibition and its implication in neurodegenerative diseases are emerging, the mechanisms by which p38 regulates TFEB-mediated autophagy and inflammatory responses in microglia remain elusive.

CMA is a pathway for selective degradation of single proteins that is mediated by binding to HSC70 (HSPA8), which recognizes a pentapeptide (KFERQ) in the substrate protein sequence. Then the substrate/chaperon complex binds the lysosome membrane-bound LAMP2A, which triggers the LAMP2A multimerization and followed lysosomal internalization of substrate proteins for degradation. The reduced neuronal CMA activity is observed in the aging brain and neurodegenerative diseases. This decline in CMA activity underpins the pathogenesis of neurodegeneration, including PD [15].

Here, we demonstrate that the p38–TFEB pathway regulates NLRP3 inflammasome degradation via CMA. We not only just identified that NLRP3 is a novel substrate for CMA, but also found that p38 inhibits TFEB activity via phosphorylation, which blocks NLRP3 degradation. Besides, our results reveal that both p38 inhibitor SB203580 and NLRP3 inhibitor MCC950 reduced microglial activation and dopaminergic neuronal loss induced by α-synuclein. All the results suggest the p38–TFEB pathway could be a potential treatment target for NLRP3 inflammasome-driven neurodegenerative diseases.

## Materials and methods

### Animal

The α-synuclein A53T-tg mice expressing mutant A53T α-synuclein (the 140 amino acid isoform) under the direction of the mouse prion protein promoter have been described previously [16]. The α-synuclein A53T-tg mice expressed mutant human A53T α-synuclein were purchased from the Model Animal Research Center of Nanjing University. The structure, location and onset of the inclusions seen in the mutant mice resemble characteristics seen in human neuronal α-synucleinopathies and these transgenic mice are generally used in the study of Parkinson's disease. Animals were individually housed under light:dark (12:12 h) cycles and provided with food and water.

### Balance beam test

Mice were placed on a narrow beam (2*3*60 cm) suspended 20 cm above soft bedding, and their movement from one end towards the other end was recorded. The number of missteps (paw faults, or slips) was scored during the trip.

### Pole test

This test was performed based on the method described by Ogawa [17] used as a measure for bradykinesia. Mice (n = 8 in each group) were placed on the top of a pole (55 cm in height, 10 mm in diameter) with a rough surface. Both the time to turn and climb down was recorded.

### Administration of the p38 inhibitor SB203580 and MCC950

All animal procedures were approved by the ethics committee of the Southern Medical University. 20 mg/kg MCC950 was used with daily oral administration [18]. Adult C57BL/6 J mice were administered intraperitoneal injections of 5 mg/kg SB203580 every other day [10]. Both MCC950 and SB203580 were dissolved in saline. In each experimental cohort, male mice were randomly matched for group assignment. α-synucleinA53T-tg mice were injected with the drug starting from 5 months of age until the end of 9 months. The midbrain and cortex of half mice were dissected and used for Western blotting analysis. Meanwhile, the other half mice were perfused with 4% paraformaldehyde and paraffinized coronal sections were processed for immunohistochemistry assay.

### Plasmids, antibody and chemicals

Plasmids: EGFP-α-synuclein A53T (40823), PHM6-α-synuclein-A53T (40825), pAAV α-synuclein WT (36055), pCDNA3 Flag p38α (20352), and pMT3-p38 (12658) were obtained from Addgene. The following antibodies were used: anti-Parkin (Cell Signaling Technologies, #4211), anti-LC3A/B (Cell Signaling Technologies, #12741), anti-beta Tubulin (Cell Signaling Technologies, #2146), anti-p38 (Abcam, ab170099), anti-p-p38tyr182 (Santa Cruz Biotechnology, Santa Cruz, CA, USA), anti-α-synuclein (Proteintech, 10842-1-AP), anti-synapsin-1 (Cell Signaling Technologies, #5297), anti-NLRP3 (Novus, NBP2-12446), anti-IL-1β (Abcam, ab9722), anti-TFEB (Proteintech, 13372–1-AP), anti-TH (Santa, sc-25269), anti-ACTB (Cell Signaling Technologies, #4970), anti-LaminB (Proteintech, 12987-1-AP), anti-SQSTM1/P62 (Proteintech, 18420-1-AP), anti-GAPDH (Proteintech, 10494-1-AP), anti-CASP1 (Proteintech, 22915-1-AP), anti-HSPA8 (Proteintech, 66442-1-Ig), anti-ASC (Cell Signaling Technologies, #67824), anti-LAMP2A (Abcam, ab18528), anti-IBA-1 (Cell Signaling Technologies, #17198), anti-GFP (Proteintech, 50430-2-AP), anti-14-3-3 (Cell Signaling Technologies, #95422), anti-FLAG (Proteintech, 66,008-3-Ig). ELISA KITs were purchased from Jiangsu Meimian industrial Co., Ltd including CASP1 (MEIMIAN, MM-0820M2) and IL-1β (MEIMIAN, MM-0040M2). SB203580 (Selleck, S1076) and MCC950 (Selleck, S7809) were purchased from Selleckchem (Houston, TX, USA). AR7 (MCE, 80306-38-3) and QX77 (MCE, HY-112483) were purchased from MCE. The siRNA for p38 was: 5′-GGUCUGUUGGAUGUGUUCAdTdT-3′. PCR primers of α-synucleinA53T-tg were: F 5′-TGTAGGCTCCAAAACCAAGG-3′, R 5′-TGTCAGGATCCACAGGCATA-3′.

### Immunohistochemistry

The paraffin sections of brain tissue were collected for routine immunohistochemistry staining for p-p38 (1:50), TH (1:100), IBA-1 (1:100), IL-1β (1:100), NLRP3 (1:100), LAMP2A (1:100) and TFEB (1:100). The area of the midbrain dopaminergic neurons from brain sections according to the anatomical position and the location of TH-positive neurons has been described previously [11].

### Transmission electron microscopy (TEM)

SN4741 cells were washed once with PBS, collected with a cell scraper. The sections of midbrain tissue were collected and washed once with cold PBS. Cell and midbrain tissue pellets were re-suspended in the fixative containing 2.5% glutaraldehyde in PBS at 4 °C. Cell and midbrain tissue pellets were fixed for 3 h. After rinsing with sodium cacodylate buffer, they were further fixed in 1% OsO_4_ in sodium cacodylate buffer on ice for 1 h and dehydrated with acetone, and then embedded in resin and polymerized at 60 °C for 48 h. Ultrathin sections were obtained on an ultramicrotome and stained with uranyl acetate and lead citrate before observation under Hitachi H-7500 TEM.

### Cell culture and transfection

SN4741 cells were cultured in DMEM medium containing 1% glucose, 100 U/ml penicillin–streptomycin, 1% l-glutamine and supplemented with 10% fetal bovine serum at 33 °C. BV2 cells were cultured in DMEM medium containing 10% fetal bovine serum (ExCell Bio, China) at 37 °C. Cells were transfected with Lipofectamine 3000 reagent (Invitrogen, Carlsbad, CA, USA) following the manufacturer's instructions.

### Western blotting and co-immunoprecipitation

Cytosolic and nuclear fractions were collected using an isolation kit (KeyGEN, Nanjing, China) following the manufacturer’s instructions. For Western blotting, cells were collected in RIPA buffer containing 20 mM Tris–HCl, pH 7.4, 150 mM NaCl and 1% Triton X-100 with protease inhibitor cocktail (Roche, Nutley, NJ, USA). The extracts were centrifuged for 10 min at 13,000 rpm at 4 °C, and the supernatants were used for immunoblot analysis. For co-immunoprecipitation, cells were centrifuged at 13,000×*g* for 10 min at 4 °C, and then the supernatant was transferred into a new tube. And then 5 μg of the corresponding first antibody was added to the samples and incubated overnight at 4 °C. The next day, 50 μl agarose beads were added to the samples and incubated 2 h at 4 °C. The immunocomplex was collected with centrifugation at 1500×*g* for 1 min at 4 °C. And washed 3 times with RIPA buffer. Proteins were eluted from beads with 2 × SDS loading buffer and subjected to immunoblot analysis. The immunoreactive bands were detected by Odyssey Infrared Imaging System. The band intensity was analyzed with Image J analysis software. Antibodies used in Western blotting are diluted by 1000, except TH (1:100) and p-p38(1:100).

### Apoptosis and cell death

Cell apoptosis of SN4741 cells was measured using a Cell Apoptosis Kit (Dojindo, Japan) according to the manufacturer’s instructions. The percent of cell apoptosis were determined by staining with 2 μM Annexin V and 2 μM propidium iodide (PI). The number of cell apoptosis was counted by flow cytometry and images of apoptosis was taken by laser scanning confocal microscopy. Cell death of primary neuron was detected by staining with PI (1 μg/ml) (KeyGEN) according to the manufacturer’s instructions. Images were captured by fluorescence microscope and analyzed by Image J analysis software.

### Measurement of mitochondrial membrane potential

The mitochondrial membrane potential in cells was assessed using tetramethylrhodamine methyl ester (TMRE; Thermo Fisher, T669). Cells were washed with 1 × PBS and then incubated with TMRE (0.1 μM) in the medium for 20 min in the dark. The intensity of fluorescence was monitored using a fluorescence microscope.

### ELISA

Tissue specimen: after cutting the specimen, weigh 0.1 g of tissue, add 9 ml of PBS, and homogenize the specimen. Centrifuge for about 20 min (12,000 rpm) and carefully collect the supernatant that used for ELISA detection following the manufacturer's instructions. The cell culture medium: the cell culture medium was centrifuged and used for ELISA detection following the manufacturer's instructions.

### Immunofluorescence

Cells were grown on a confocal dish for 2 days, and then cells were washed with PBS three times, then fixed with 4% paraformaldehyde for 15 min at room temperature. After that, cells were permeabilized with frozen methanol for 10 min at − 20 °C and blocked in 5% BSA for 30 min. And then samples were incubated with primary antibody (1:100–1:200) in 5% BSA overnight at 4 °C, and then incubated with secondary antibody (1:100) in 5% BSA for 1 h at room temperature. An Olympus FV1000 confocal microscope with a × 100 objective was used for image capture. The co-localization signal was analyzed with Image Pro Plus software.

### Statistical analysis

All data of experiments were analyzed with GraphPad Prism 7 software (La Joya, Ca, USA). Data from the Western blot and immunohistochemistry analysis were performed with the t-test for two groups or ANOVA with Tukey’s post-test (GraphPad Software) for multiple groups. Data from the balance beam test, pole test, transmission electron microscopy, immunofluorescence, were analyzed by two-way ANOVA followed by Tukey’s post-test. All values are expressed as the mean ± SEM, and p values < 0.05 were considered statistically significant. All experiments were repeated at least 3 times.

## Results

### NLRP3 inflammasome is activated in α-synucleinA53T-tg mice

**Fig. 1:**
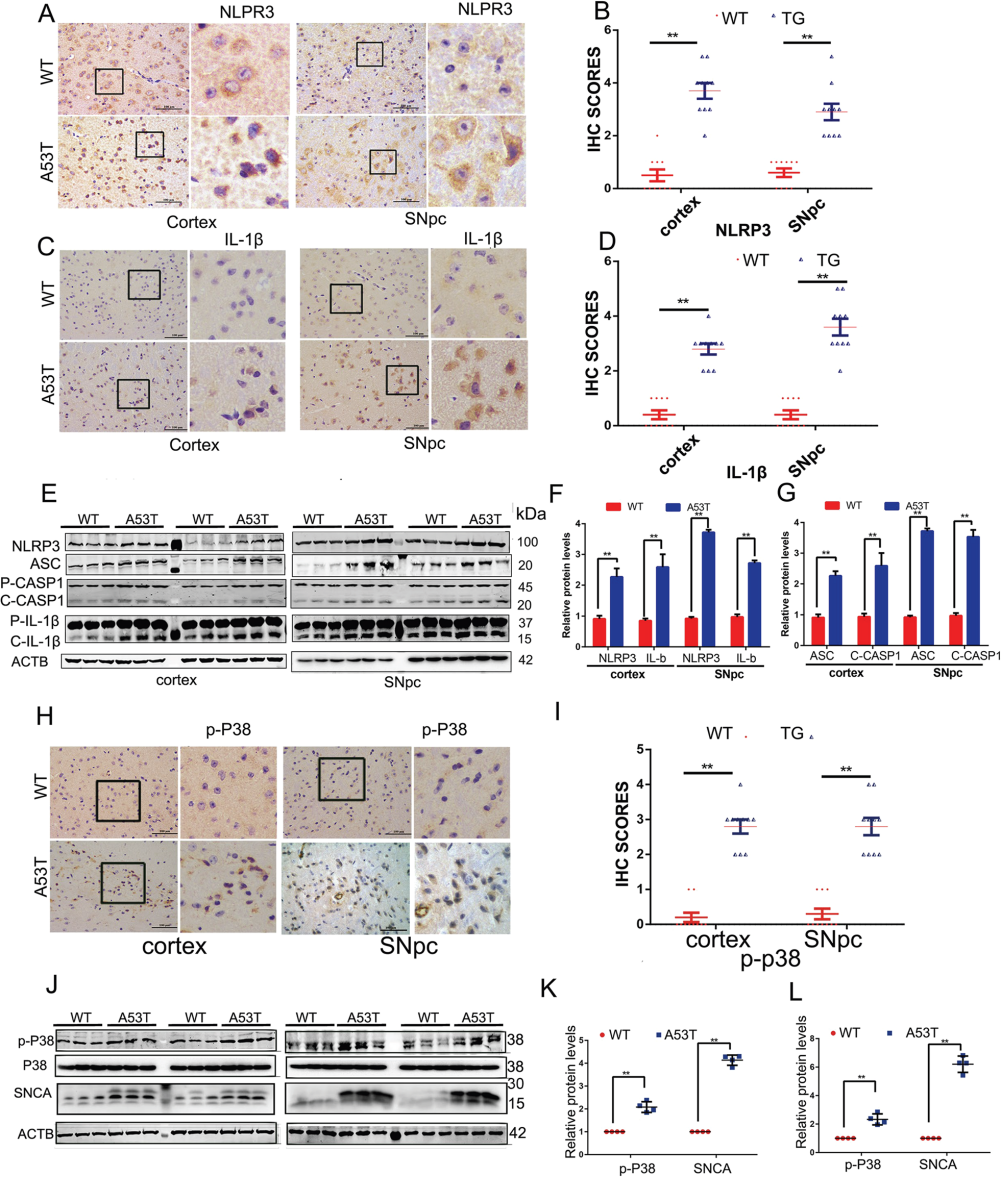
NLRP3 inflammasome is activated in the α-synucleinA53T-tg mice. **A** Immunohistochemistry (IHC) demonstrating increased NLRP3 protein levels in the cortex and SNpc of 9–month-old α-synucleinA53T-tg mice. Scale bars, 100 μm. **B** Statistical analysis of the scores of NLRP3 staining between α-synucleinA53T-tg and wild-type mice. *p < 0.05 (Student’s t-test). **C** IHC demonstrating increased IL-1β protein levels in the cortex and SNpc of 9–month-old α-synucleinA53T-tg mice. Scale bars, 100 μm. **D** Statistical analysis of the scores of IL-1β staining between α-synucleinA53T-tg and wild-type mice. *p < 0.05 (Student’s t-test). **E**–**G** Cell lysates from the cortex and SNpc of 9-month-old α-synucleinA53T-tg or wild-type mice were immunoblotted. The protein levels of NLRP3, ASC, cleaved CASP1, IL-1β were statistically analyzed in **F** and **G**. Mean ± SEM, n = 6, *p < 0.05 (Student’s t-test). **H**, **I** IHC demonstrating increased phosphorylated p38 levels in the cortex and SNpc of 9-month-old α-synucleinA53T-tg mice. Scale bars, 100 μm. **I** Statistical analysis of the scores of phosphorylated p38 between α-synucleinA53T-tg and wild-type mice. *p < 0.05 (Student’s t-test). **J**, **K** Lysates from the cortex and SNpc of mice were immunoblotted using the indicated antibodies. The protein levels of phosphorylated p38 and α-synuclein were statistically analyzed in D and E. Mean ± SEM, n = 6, *p < 0.05 (Student’s t-test)

In order to investigate the effects of α-synuclein accumulation and related toxicity, we used α-synuclein A53T transgenic mice (α-synucleinAA53T-tg mice). The accumulation of α-synuclein (SNCA) was identified in both the SNpc and the cortex at 9 months of age (Additional file 1: Fig. S1A). To determine the effect of α-synuclein on NLRP3 inflammasome activation, we measured the expression of core components of this multiprotein complex of the canonical inflammasome in α-synucleinA53T-tg mice by IHC and Western blotting. In the brain sections of the SNpc and the cortex, NLRP3 inflammasome activation was remarkably intensified, as evidenced by increased NLRP3 expression as well as over-production of IL-1β (Fig. 1A–G). Besides, microglial activation, as assessed morphologically by immunohistochemistry with the classic antibody specific for Iba-1, microglia were activated in α-synucleinA53T-tg mice brain, both of the SNpc and the cortex (Additional file 1: Fig. S1B). We also detected the dynamic changes in NLRP3 inflammasome activation through measuring the co-staining between Iba1 and NLRP3 from 3 to 9 months old in Additional file 1: Fig. S1C, which showed that the activity levels of NLRP3 in microglia increases with months. To further confirm this result, PD patients’ serum was assayed by ELISA. Consistently, over-production of IL-1β and IL-18 was found in PD patients’ serum (Additional file 1: Fig.S1D–E). Collectively, these data suggest that the NLRP3 inflammasome is activated in the pathological process of PD.

### NLRP3 inflammasome activation is suppressed by p38 inhibitor SB203580

To examine whether p38 is involved in the NLRP3 inflammasome activation in PD, we first investigated the levels of p38 phosphorylation by IHC. Results showed that compared with wild-type mice, p38 was activated in both the SNpc and the cortex of α-synucleinA53T-tg mice at 9 months of age (Fig. 1H, I). Western blotting also identified increased phosphorylated p38 and α-synuclein accumulation in the α-synucleinA53T-tg mice (Fig. 1J–L).

**Fig. 2:**
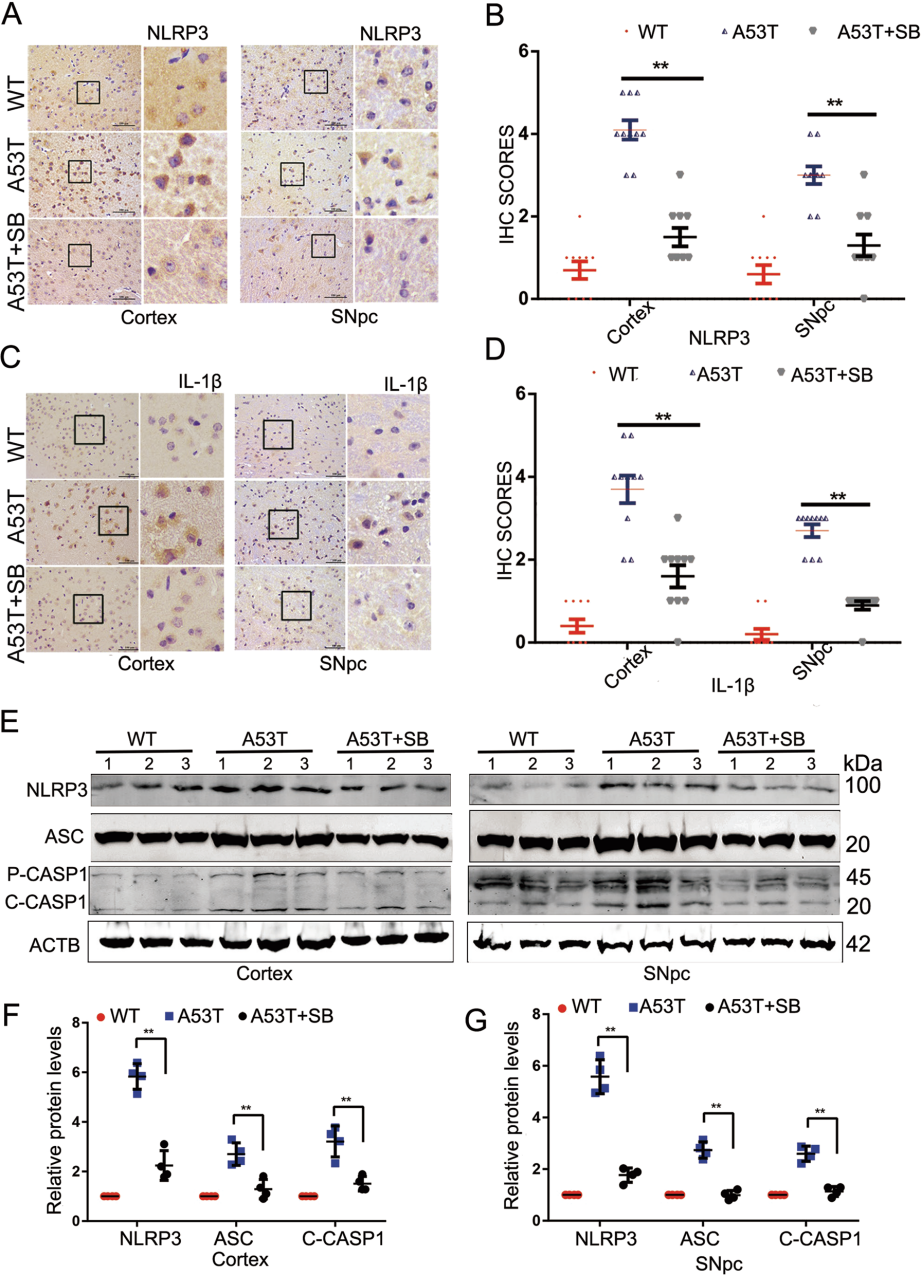
p38 inhibitor SB203580 inhibits the activation of NLRP3 inflammasome. **A** IHC demonstrating SB203580 reduced NLRP3 protein levels in the cortex and SNpc of 9-month-old α-synucleinA53T-tg mice. Scale bars, 100 μm. **B** Statistical analysis of the scores of NLRP3 staining between α-synucleinA53T-tg and wild-type mice. *p < 0.05. **C** IHC demonstrating SB203580 reduced IL-1β protein levels in the cortex and SNpc of 9-month-old α-synucleinA53T-tg mice. Scale bars, 100 μm. **D** Statistical analysis of the scores of IL-1β staining between α-synucleinA53T-tg and wild-type mice. *p < 0.05. **E**–**G** Cell lysates from the cortex and SNpc of mice were immunoblotted using the indicated antibodies. The protein levels of NLRP3, ASC, cleaved CASP1 were statistically analyzed in **F** and **G**. *p < 0.05

To assess whether p38 mediates NLRP3 activation, α-synucleinA53T-tg mice were injected with p38 inhibitor SB203580 starting at the age of 5 months until the end of 9 months. Then, we analyzed NLRP3 and IL-1β by IHC. The results showed that SB203580 significantly reduced the levels of NLRP3 and IL-1β both in SNpc and cortex of α-synucleinA53T-tg mice (Fig. 2A–D). Furthermore, the increased protein levels of NLRP3, ASC, cleaved CASP1, and cleaved IL-1β in α-synucleinA53T-tg mice (Fig. 2E–G). Previous work identifies a key role of microglia and NLRP3 inflammasome activation in the pathogenesis of neurodegeneration [19–21]. Therefore, we use the classic microglial cell line BV2 cells to explore the potential mechanisms of NLRP3 activity. The increased protein levels of NLRP3, ASC, cleaved CASP1, and cleaved IL-1β in BV2-A53T cells were abolished after SB203580 treatment (Additional file 2: Fig. S2C, D). Also, we have detected the Caspase 1 activity as well as mature IL-1β using ELISA in the tissue homogenates, and results revealed that SB203580 could decrease the levels of Caspase 1 activity and mature IL-1β in α-synucleinA53T-tg mice (Additional file 2: Fig. S2E, F). Of note, SB203580 did not affect the transcription of α-synuclein (Additional file 2: Fig. S2G). Together, these data suggested that p38 inhibitor SB203580 reduced neuroinflammation caused by α-synuclein accumulation.

### SB203580 induces NLRP3 degradation via chaperone-mediated autophagy

**Fig. 3:**
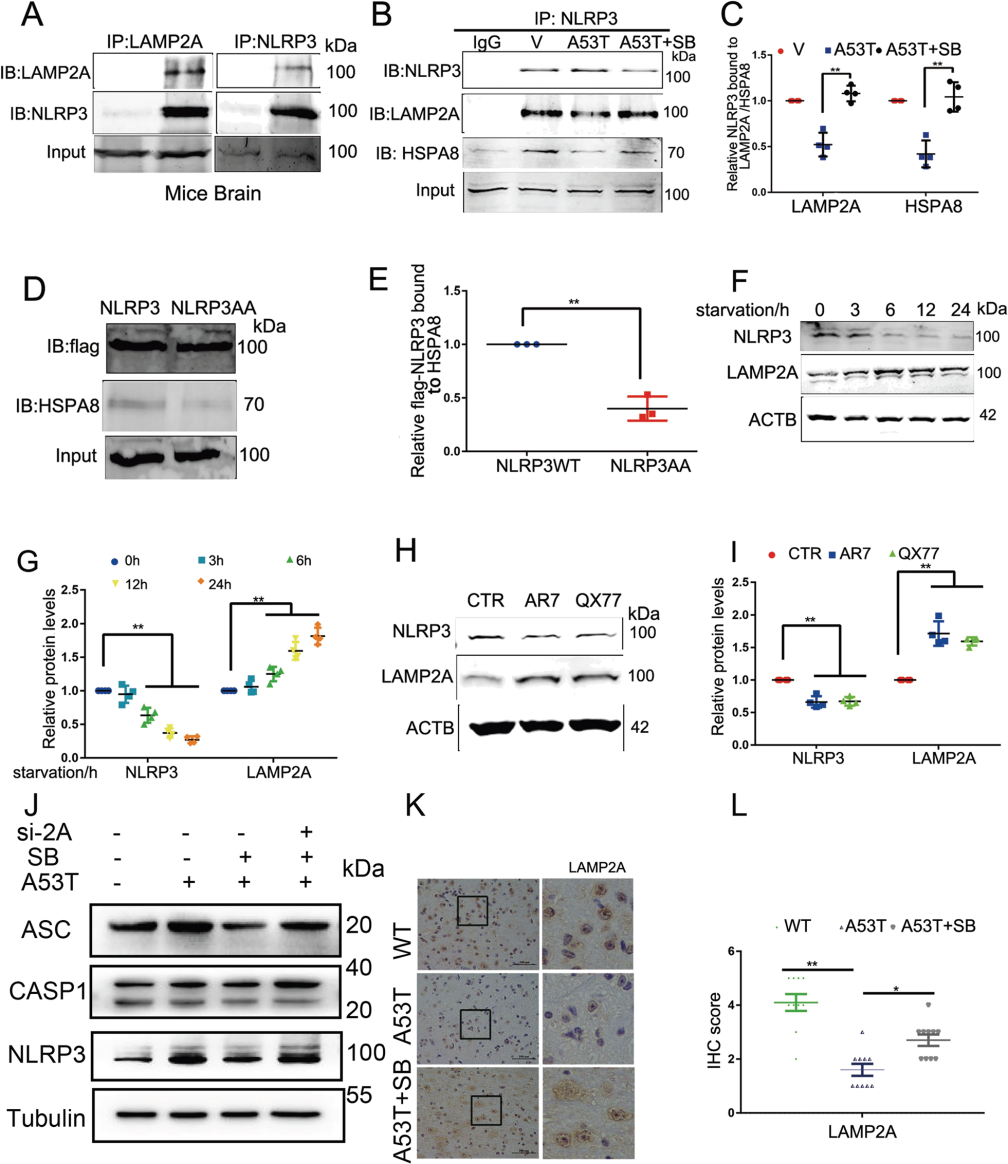
NLRP3 is degraded through CMA. Brain lysates from mice were used for IP using anti-NLRP3 or anti-LAMP2A antibody. **B**, **C** Cell lysates from BV2 cells were used for IP with NLRP3. SB203580 increased the NLRP3/LAMP2A and NLRP3/HSPA8 interaction and showed in **C**. **D**, **E** Cell lysates from BV2 cells were used for IP with anti-Flag antibody. Wild-type NLRP3-Flag but not NLRP3AA-Flag was coimmunoprecipitated with HSPA8. **F**, **G** Cell lysates from BV2 cells were immunoblotted to determine the levels of LAMP2A and NLRP3 after starvation. The protein levels of LAMP2A and NLRP3 were statistically analyzed in **G**. Mean ± SEM, n = 3, *p < 0.05. **H**, **I** Cell lysates from BV2 cells were immunoblotted to determine the levels of LAMP2A and NLRP3 after treatment with AR7 and QX77. The protein levels of LAMP2A and NLRP3 were statistically analyzed in **L**. Mean ± SEM, n = 3, *p < 0.05. **J** BV2 cells were treated with LAMP2A siRNA. Cell lysates from BV2 cells were immunoblotted to determine the levels of NLRP3, ASC and CASP1 after treatment with SB203580. LAMP2A siRNA effectively blocked SB203580-induced NLRP3 degradation. **K**, **L** IHC demonstrating decreased LAMP2A protein levels were rescued by SB203580 in the SNpc of α-synucleinA53T-tg mice. Statistical analysis of the scores of LAMP2A staining is shown in **L**. *p < 0.05

The activation of NLRP3 inflammasome in cells is tightly regulated. Excessive activation of the NLRP3 inflammasome is involved in the pathological process of various diseases including PD. To test whether CMA degrades NLRP3, the interaction between NLRP3 and LAMP2A was examined by immunoprecipitation in brain tissues. The results showed that LAMP2A interacts with NLRP3 in vivo (Fig. 3A). Moreover, we found that SB203580 enhanced the NLRP3/LAMP2A and NLRP3/ HSPA8 interaction in BV2 cells (Fig. 3B, C).

In CMA, the substrate usually contains a KFERQ-like pentapeptide consensus sequence that is recognized by HSPA8 (HSC70). Inspection of the amino acid sequence of mouse NLRP3, four KFERQ-like motifs (355LEKLQ359, 603QIRLE607, 795QKLVE799 and 989EVLKQ993), were revealed (Additional file 2: Fig. S2H). To determine whether NLRP3 is a bona fide substrate for CMA, the first two amino acids of the mouse NLRP3 KFERQ-like sequence were mutated to alanine (NLRP3AA). The results showed that mutation of the KFERQ-like sequence of NLRP3 abolished its interaction with HSPA8 (Fig. 3D, E).

To further assess whether CMA is involved in the regulation of NLRP3, we treated BV2 cells and primary microglia with serum deprivation, AR7 (10 µM, 24 h) or QX77 (10 µM, 24 h), the latter two chemicals are well-known CMA agonists. The results showed that both serum deprivation and CMA agonists increased the levels of LAMP2A and reduced NLRP3 (Fig. 3F–I and Additional file 2: Fig. S2 I–M). To further confirm SB2035802 induced NLRP3 degradation through CMA, we treated BV2 cells with LAMP2A siRNA. As Fig. 3 shows, LAMP2A siRNA effectively blocked SB203580-induced NLRP3 degradation (Fig. 3J). Moreover, the level of LAMP2A in the SNpc of mice was analyzed. Treatment with the p38 inhibitor SB203580 elevated the level of LAMP2A, a key receptor for CMA (Fig. 3K, L).

### SB203580 activates TFEB-mediated autophagy

**Fig. 4:**
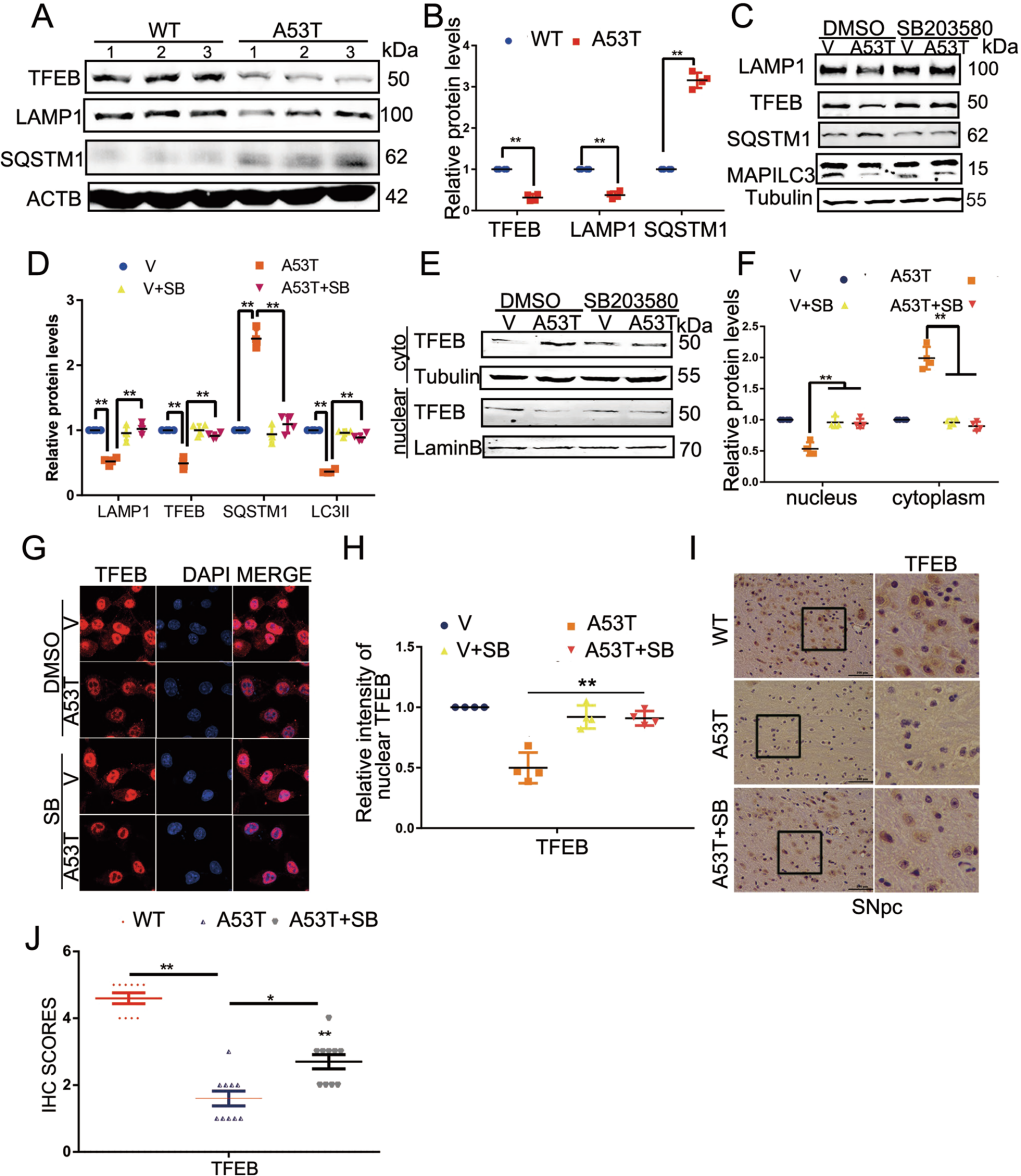
SB203580 activates autophagy through TFEB. **A**, **B** Lysates from the SNpc of mice were immunoblotted using the indicated antibodies. The protein levels of TFEB, LAMP1 and SQSTM1 (P62) were statistically analyzed in **B**. Mean ± SEM, n = 6, *p < 0.05. **C**, **D** Cell lysates from BV2 cells were immunoblotted to determine the levels of LAMP1, MAP1LC3, and SQSTM1(P62) and presented in **D**. Mean ± SEM, n = 3, *p < 0.05. **E**, **F** BV2 cells were subjected to subcellular fractionation to determine nuclear translocation TFEB in α-synuclein A53T BV2 cells and presented in **F**, *p < 0.05. **G**, **H** Subcellular localization of TFEB was analyzed by confocal microscopy and presented in **H**. Scale bars, 10 μm. *p < 0.05. **I**, **J** IHC demonstrating decreased TFEB protein levels were rescued by SB203580 in the SNpc of α-synucleinA53T-tg mice. Scale bars, 100 μm. Statistical analysis of the scores of TFEB staining were shown in **J**. *p < 0.05

Our data in Figs. 2 and 3 demonstrate that p38 mediates the NLRP3 inflammasome activation in BV2 cells and α-synucleinA53T-tg mice, as evidenced by increased levels of NLRP3, cleaved CASP1, and IL-1β. Furthermore, in Fig. 4, we showed that p38 regulates NLRP3 turnover through CMA. All these prompt us to examine the role of p38 in autophagy regulation, which may subsequently contribute to the NLRP3 inflammasome activation [24]. TFEB is a master protein for lysosomal biogenesis. In SNpc sections from α-synucleinA53T-tg mice, the total levels of TFEB and LAMP1 were decreased, while the SQSTM1/p62 level was increased (Fig. 4A, B). To determine whether α-synuclein aggregation affects the TFEB nuclear translocation that regulates the transcription of autophagy-related genes, subcellular localization of TFEB was examined by subcellular fractionation. The result showed that the nuclear TFEB level decreased in SNpc sections of α-synuclein A53T-tg mice (Additional file 3: Fig. S3A, B).

To examine whether p38 activation is involved in TFEB-mediated autophagy in the PD model, we treated α-synuclein-A53T overexpressing BV2 cells with p38 inhibitor SB203580 (10 μM, 24 h). The results showed that p38 inhibitor SB203580 significantly elevates TFEB-mediated autophagy, as evidenced by the increased LAMP1, MAP1LC3/LC3, and the decrease of SQSTM1 (Fig. 4C, D). Furthermore, SB203580 prompted TFEB nuclear translocation from the cytoplasm (Fig. 4E–H). Meanwhile, SB203580 enhanced the biogenesis of lysosomes (Additional file 3: Fig. S3 C, D). Besides, SB203580 also increased the level of TFEB in SNpc of mice (Fig. 4I, J). The EM images of the lysosomal morphology and the autophagy-related proteins LC3 and P62 in brain tissue were measured (Additional file 3: Fig. S3E–F). All these data suggested that SB203580 promotes TFEB-mediated autophagy.

### p38 interacts with and phosphorylates TFEB at serine 211

**Fig. 5:**
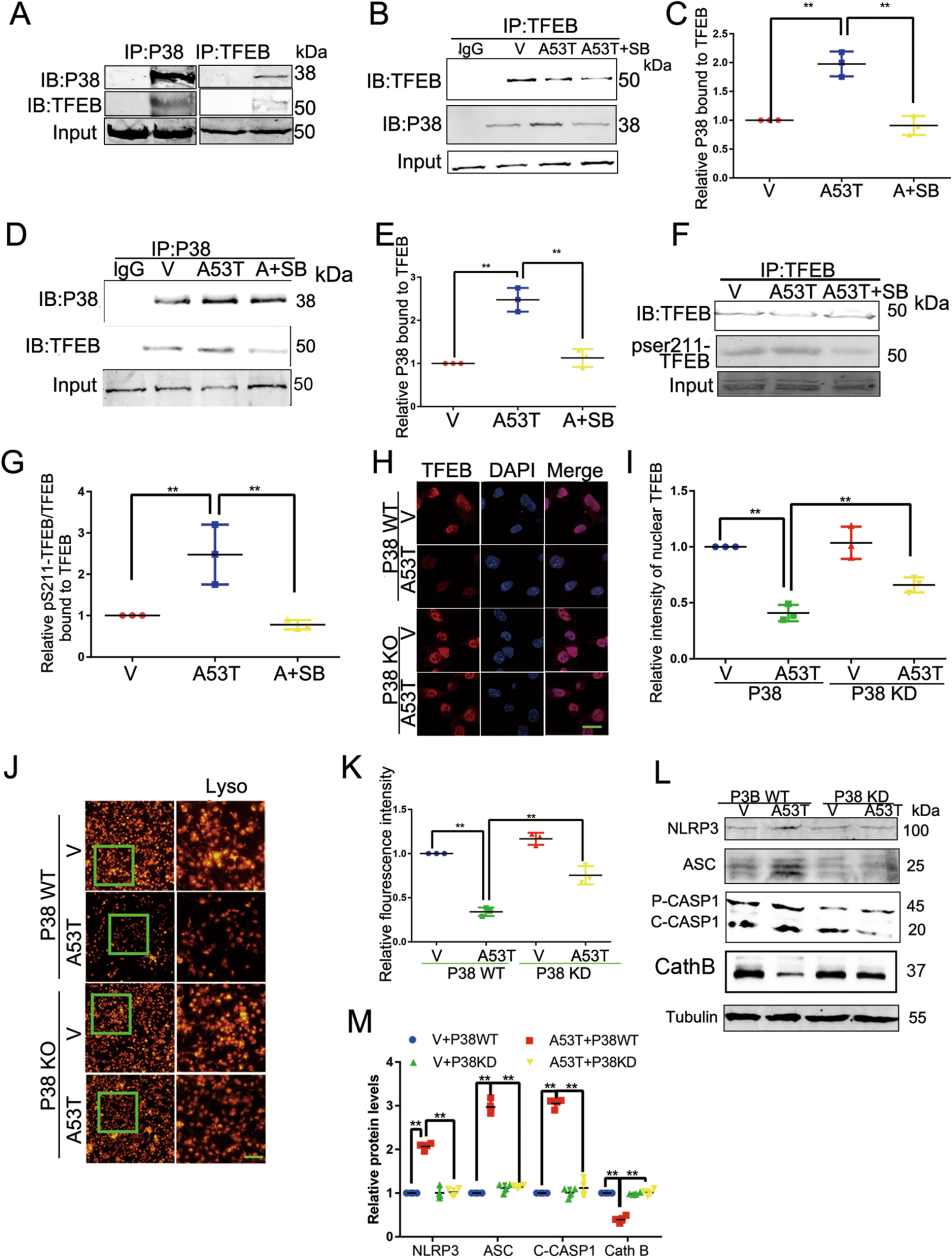
p38 interacts with and phosphorylates TFEB at serine 211. **A** Lysates from mouse brain were used for IP with anti-TFEB antibody or anti-p38 antibody. **B**, **C** Cell lysates from BV2 cells were used for IP with anti-TFEB antibody. SB203580 reduced the TFEB/p38 interaction. *p < 0.05. **D**, **E** Cell lysates from BV2 cells were used for IP with anti-p38 antibody. SB203580 reduced the TFEB/p38 interaction and shown in **E**. Mean ± SEM, n = 3. *p < 0.05. **F**, **G** Cell lysates from BV2 cells were used for IP with anti-GFP antibody. SB203580 reduced the TFEB–GFP/14-3-3 interaction. Mean ± SEM, n = 3. *p < 0.05 (**H**, **I**). Subcellular localization of TFEB was analyzed by confocal microscopy and shown in I. Scale bars, 10 μm. Mean ± SEM, n = 3, *p < 0.05. **J**, **K** BV2 cells were labeled with lysosome tracker and visualized lysosome biogenesis under a microscope and shown in **K**. Scale bars, 100 μm. Mean ± SEM, n = 10, *p < 0.05. **L**, **M** Cell lysates from BV2 cells were immunoblotted to determine the levels of NLRP3, ASC, cleaved CASP1, CathB and shown in **M**. Mean ± SEM, n = 3, *p < 0.05

Next, we aimed to elucidate the mechanism by which p38 regulates TFEB-mediated autophagy. To evaluate whether TFEB could be a substrate for p38, we first detected the interaction between p38 and TFEB by co-immunoprecipitation. As expected, immunoprecipitation analysis showed that p38 interacted with TFEB both in BV2 cells and mice brains (Fig. 5A–E). Of note, SB203580 reduced the p38/TFEB interaction that was enhanced by α-synuclein A53T aggregation (Fig. 5B–E).

To identify the regulatory function of p38 on TFEB, we examined the role of p38 on the interaction between TFEB and14-3-3 proteins. As phosphorylation of TFEB at serine 211 (p-Ser TFEB211) by mTORC1 has been proved to promote TFEB binding to 14-3-3, we detected the level of TFEB phosphorylation with a phospho-Ser211-specific antibody. It showed that SB203580 reduced TFEB phosphorylation at serine 211 (Fig. 5F, G). To explore the mechanism by which p38 regulates TFEB’s function, we analyzed the effect of overexpression kinase-dead p38 mutant on TFEB’s nuclear translocation. Compared with p38 WT, kinase-dead p38 overexpression increased the nuclear translocation of TFEB (Fig. 5H, I) and enhanced the biogenesis of lysosomes (Fig. 5J, K). Meanwhile, kinase-dead p38 overexpression declined the levels of NLRP3, ASC, C-CASP1, Cathepisn B (Cath B), pSerTFEB 211 and the IL1βand IL-18 (Fig. 5L, M and Additional file 3: Fig S3G-I). To further confirm the effects of p38, we analyzed the effect of p38 siRNA. As Additional file 4: Fig. S4 shows, p38 siRNA increased the biogenesis of lysosome (Additional file 4: Fig. S4A–B). Also, p38 siRNA decreased the levels of NLRP3, C-CASP1 detected by WB (Additional file 4: Fig. S4C, D). These results supported that p38 activation suppresses TFEB function via phosphorylating TFEB at serine 211 and thus inhibiting TFEB nuclear translocation.

These findings including the interaction between p38 and TFEB, the inhibitory effects of p38 inhibitor and the activation of NLRP3 inflammasome related to autophagy, raise the possibility that p38 may curb TFEB to increase NLRP3 inflammasome activation. To test this hypothesis, we first overexpressed TFEB in α-synuclein-A53T BV2 cells. The results showed that TFEB overexpression reduced NLRP3 and cleaved CASP1 (Additional file 4: Fig. S4E, F). To examine whether p38 activates NLRP3 via regulating TFEB, we compared the effects of SB203580 treatment, TFEB knockdown, and both SB203580 treatment and TFEB knockdown in α-synuclein-A53T BV2 cells. Remarkably, the overactivation of NLRP3 inflammasome and cleaved CASP1 resulting from α-synuclein-A53T was almost completely reversed by SB203580, while TFEB knockdown eliminated the effect of SB203580 (Additional file 4: Fig. S4G, H). Similarly, the effect of SB203580 on IL-1β, IL-18 was abrogated in α-synuclein-A53T BV2 cells with TFEB knockdown (Fig. 4I, J). To further test whether the autophagy/lysosome pathway is responsible for p38 inhibition-induced NLRP3 degradation, we treated α-synuclein-A53T BV2 cells with autophagy/lysosome inhibitor CQ (10 µM, 4 h). The results showed that CQ blocked SB203580-induced NLRP3 degradation (Additional file 4: Fig. S4K, L), which further supported that p38 activates NLRP3 inflammasome via disturbing TFEB-mediated autophagy.

### α-Synuclein-induced NLRP3 activation in microglia promoted dopaminergic neuronal loss

To examine whether microglial activation is responsible for the death of dopaminergic neurons, SN4741 cells were treated with a conditioned medium from microglia. After appropriate treatments, cell death in SN4741 cells was determined by flow cytometry. As Additional file 5: Fig. S5A–B shows, conditioned medium from α-synuclein-A53T BV2 cells caused the dopaminergic neuronal loss, while the NLRP3 inhibitor MCC950 (10 µM, 12 h) and the p38 inhibitor SB203580 effectively eliminated the effect.

**Fig. 6:**
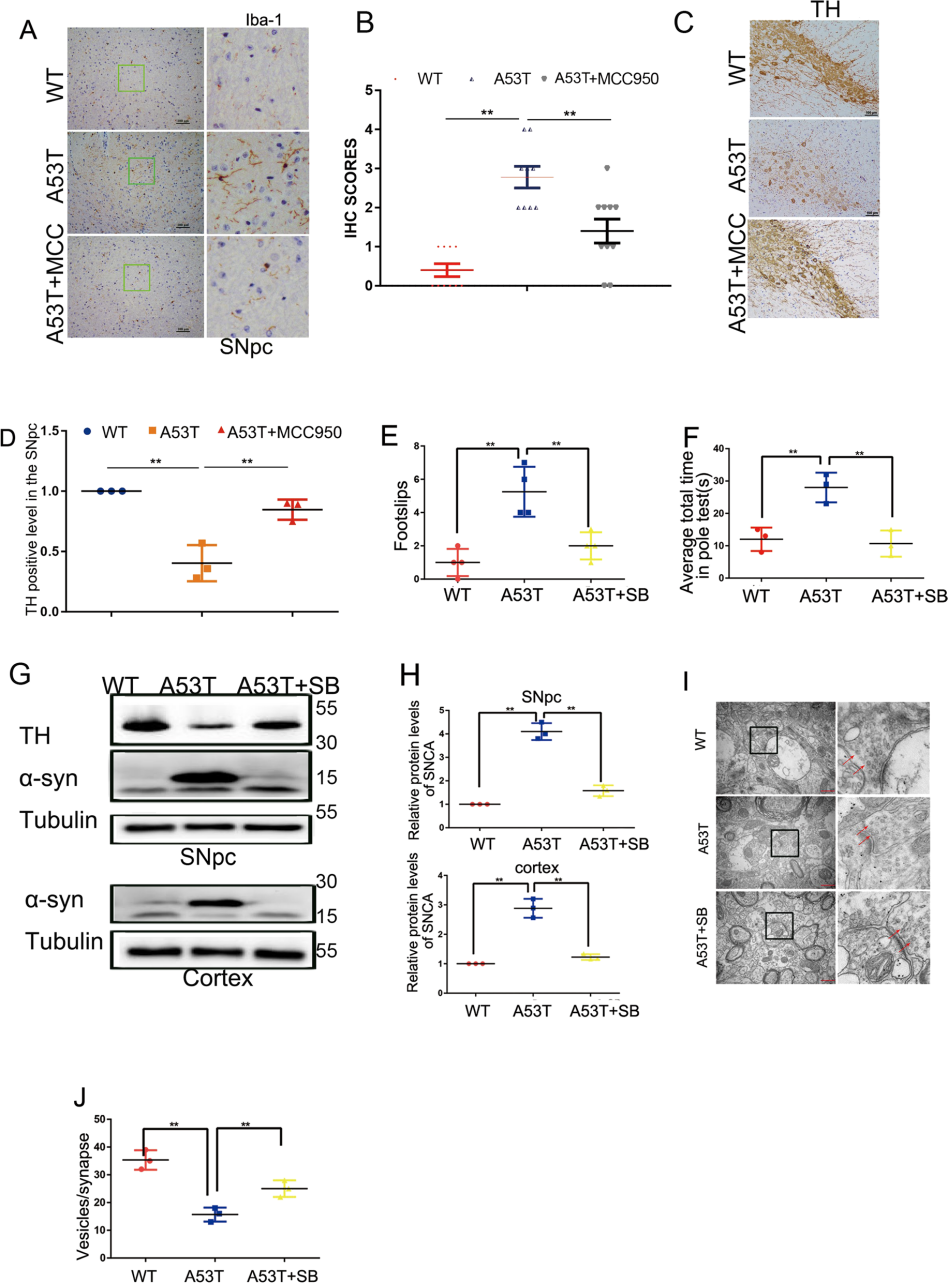
α-Synuclein-induced NLRP3 activation in microglia promoted dopaminergic neuronal loss. **A**, **B** Immunohistochemistry (IHC) demonstrating the levels of IBA-1 in the SNpc of 9 months of wild type, α-synuclein A53T-tg and α-synuclein A53T-tg mice treated with MCC950. Statistical analysis of the scores of IBA-1 staining is shown in B. Scale bars, 100 μm. Mean ± SEM, n = 3. *p < 0.05. **C**, **D** Immunohistochemistry (IHC) demonstrating the levels of TH in the SNpc of 9 months of wild type, α-synuclein A53T-tg and α-synuclein A53T-tg mice treated with MCC950. Statistical analysis of the levels of TH staining is shown in D. Scale bars, 100 μm. Mean ± SEM, n = 3. *p < 0.05. **E** Balance beam foot slips were quantified after PBS or SB203580 injection. **F** Results of the pole test were quantified after PBS or SB203580 injection. **G**, **H** Cell lysates from the SNpc and cortex of mice were immunoblotted using the α-synuclein and TH antibody. Mean ± SEM, n = 6, *p < 0.05. **I**, **J** TEM was used to examine the number of synapses. The provided scale bar in merge the image represented 500 nm

To address whether NLRP3 activation is involved in dopaminergic neuronal loss in vivo, α-synucleinA53T-tg mice were fed with the NLRP3 inhibitor MCC950 starting at the age of 5 months until the end of 9 months. Interestingly, MCC950 significantly inhibited the activation of microglia in α-synucleinA53T-tg mice, which suggests that NLRP3 plays a key role in the activation of microglia in PD (Fig. 6A, B). Then, we analyzed the expression of TH in SNpc. And results showed that MCC950 significantly reduced the levels of dopaminergic neuronal loss in SNpc of α-synucleinA53T-tg mice (Fig. 6C, D). Furthermore, the balance beam test and pole test showed that MCC950 treatment significantly improved motor activity in α-synucleinA53T-tg mice (Additional file 5: Fig. S5C, D).

## SB203580 plays a protective role in α-synucleinA53T-tg mice

The above data showed that the p38-TFEB pathway is involved in NLRP3 inflammasome activation, which prompts us to test whether p38 inhibitor SB203580 has a protective role in α-synucleinA53T-tg mice. Wild type and α-synucleinA53T-tg mice were treated with or without the p38 inhibitor SB203580. Balance beam test and pole test showed that SB203580 treatment significantly improved the motor activity of α-synucleinA53T-tg mice (Fig. 6E, F). In addition, abnormal accumulation of α-synuclein and decreasing TH could be alleviated after p38 inhibition (Fig. 6G, H). Synapse loss correlates with cognitive impairment in AD and PD [22, 23]. Actually, SB203580 rescues the reduced level of synapsin-1 (SYN1, the presynaptic protein) in α-synucleinA53T-tg mice (Additional file 5: Fig. S5E, F) that confirmed the protective role of p38 inhibition. Under TEM, α-synucleinA53T-tg mice have fewer synaptic vesicles, while SB203580 treatment partially restores synaptic vesicle loss (Fig. 6I, J). Taken together, these data indicated that p38 inhibition provides protection in α-synucleinA53T-tg mice.

## Discussion

PD is a common, age-related neurodegenerative disease afflicting more than 2–3% of people over 65 and more than 4% of the population by the age of 85. The progressive loss of dopaminergic neurons in the substantia nigra eventually results in motor alterations of PD [24]. The precise mechanism underlying the pathogenesis of PD is not yet been fully elucidated. Previous studies have shown the defect of the autophagy–lysosomal pathway (ALP) and neuroinflammation are contributors to PD pathophysiology [8, 11]. Accumulating evidence suggests that immune signaling cascades are regulated by autophagy, while the mechanisms that modulate the inflammasome activation process are still unclear. In our study, NLRP3 was identified as a novel chaperone-mediated autophagy substrate and activation of CMA promotes NLRP3 degradation and thereby inhibits the over-production of proinflammatory cytokines in microglial cells. Moreover, we demonstrated that p38 activation caused by accumulated α-synuclein inhibits TFEB-mediated CMA, which promotes NLRP3 inflammasome activation. Furthermore, we found that both p38 and NLRP3 inhibitors can reduce aggregated α-synuclein-induced microglia activation and dopaminergic neuronal loss. Therefore, p38–TFEB pathways regulate NLRP3 degradation via CMA in aggregated α-synuclein-induced microglia activation promoting dopaminergic neuronal loss.

Chronic neuroinflammation in PD has been intensively investigated in the past decade, but the precise origin of the CNS inflammation response remains unclear. Evidence found that neuroinflammatory response is a key factor in the pathological process of PD [25, 26]. Activated microglia and increased levels of proinflammatory factors were identified in postmortem analysis and PD models. α-Synuclein abnormal aggregation can appear in neurons and glia, which not only disrupt neuronal function, but also causes activation of microglia that increase phagocytic activity and pro-inflammatory cytokines production [27]. It is still controversial whether α-synuclein accumulation can trigger microglia responses and pathological reactions. Our results not only identified that NLRP3 was a novel CMA substrate, but also revealed that p38 activation caused by pathologic α-synuclein inhibits TFEB activity that blocked CMA, and then decreased NLRP3 degradation in microglia. Studies found that α-synuclein abnormal aggregation activated microglia, which led to the death of dopaminergic neurons in the substantia nigra of the midbrain [28]. Toll-like receptor 2 (TLR2) of microglia was identified as the receptor for secreted α-synuclein, which transports α-synuclein into the microglial cytoplasm [27]. Overexpression of α-synuclein increases microglial activation in vivo. However, α-synuclein internalization still appears when TLR2 is deficient, suggesting α-synuclein uptake relies on multiple receptor systems [28].

The activation of the NLRP3 inflammasome usually requires upregulation of the transcription of NLRP3 and pro-IL-1β by NF-κB. However, recent studies report that NLRP3 inflammasome can be regulated independently of transcription. For instance, lys-63-specific deubiquitinase BRCC36 promotes NLRP3 deubiquitination and facilitates NLRP3 activation. Previous studies showed that NLRP3 inflammasome is activated by pathologic α-synuclein. In this work, we revealed that the degradation of NLRP3 through CMA is an essential event for the regulation of NLRP3 inflammasome. Our study demonstrated that the p38–TFEB pathway regulates the NLRP3 inflammasome in microglia which is involved in the pathological process of PD. Importantly, the protective effect of p38 inhibition on anti-inflammation and autophagy enhancement, which has been shown to ameliorate the pathological symptoms of neurodegeneration diseases. Several studies revealed that p38 activation is closely connected to autophagy dysfunction. Mao and colleagues identified that p38 regulates macroautophagy and CMA via phosphorylating ULK and LAMP2A, respectively [8]. Besides, the p38 MAPK–Parkin signaling pathway regulates mitochondrial homeostasis and neuronal degeneration pathway in the published paper [11]. Yan et al. reported that neurotransmitter dopamine inhibits NLRP3 inflammasome via dopamine D1 receptor, which shows that the nervous system and neurotransmitters play a role in neuroinflammation [29]. And recent studies have shown that autophagy plays a key role in inflammasome regulation [30]. Notably, autophagy dysfunction disrupts cell homeostasis thus inducing excessive activation of inflammasomes. As a member of the MiT family of transcription factors, TFEB binds to the promoter motif of autophagy, lysosomal biogenesis-related genes, promote the transcription of those genes. The transcriptional activity of TFEB is controlled by post-translational modification such as phosphorylation, acetylation. The mTORC1 kinase phosphorylates S122, S142 and S211 in the TFEB protein, regulating the TFEB subcellular localization. TFEB can be phosphorylated by AKT at residues S467 leading to cytoplasmic retention. On the contrary, calcineurin de-phosphorylating TFEB results in TFEB nuclear translocation. PKCβ phosphorylates S462, S463, S467 and S469, which does not affect TFEB subcellular localization but its protein stability. Zhang et al. reported that acetylation of TFEB at residues K91, K103, K116 and K430 determined its transcriptional activity [31]. In short, research indicates the kinases phosphorylate TFEB and subcellular localization. Kim SH et al. show that ezetimibe blocked the NLRP3 inflammasome–IL1B pathway by autophagy induction through AMPK phosphorylation, activation and nuclear translocation of TFEB [32]. The evidence show NLRP3 inflammasome is activated in the pathological process of PD. Yao et al. reported that MiR-124 inhibited the secretion of proinflammatory mediators by promoting autophagy in PD [33]. TFEB phosphorylation and resultant activation of NLRP3 inflammasome in PD-associated neuroinflammation are still unclear. This study provided evidence showing that p38/TFEB-mediated CMA regulates the NLRP3 inflammasome. Although we identified the role of p38 in the activation of NLRP3 inflammasome in α-synucleinA53T mice, the mechanism may be applicable in other neurodegenerative diseases.

## Conclusions

In conclusion, we uncovered a novel NLRP3 degradation pathway by CMA in microglia, which is negative regulation by p38 through direct phosphorylating TFEB at ser211, inhibits proteins expression of autophagy. Moreover, p38 inhibitor SB203580 provided protection, via enhancing TFEB-mediated CMA and reducing NLRP3 inflammasome activation. Given that neuroinflammatory response is deeply involved in neurodegenerative diseases, the mechanisms we identified here shed new light on the neurodegenerative process. In addition, the p38–TFEB-mediated CMA may be a potential therapeutic target for PD.

## Supplementary Information

**Additional file 1: Fig S1.** (A) Immunohistochemistry (IHC) demonstrating increased α-synuclein in the cortex and SNpc of 9 months α-synuclein A53T-tg or wild-type mice. Statistical analysis of the average score of α-synuclein staining between α-synuclein A53T-tg and wild-type mice. *p<0.05 (Student’s t-test). (B) Immunohistochemistry (IHC) demonstrating increased IBA-1 in the cortex and SNpc of 9 months α-synuclein A53T-tg or wild-type mice. (C) The co-staining between Iba1 and NLRP3 was detected by IF, which the dynamic changes of NLRP3 inflammasome activation in microglia from 3 months to 9 months of age. (D, E) Levels of IL-1β and IL-18 in serum were assessed by ELISA assay in 10 patients suffering from PD and 10 control. Data were performed using the Student’s unpaired t-test.

**Additional file 2: Fig S2** Lysates from the cortex and SNpc of mice were immunoblotted using the indicated antibodies. The protein levels of p-p65 were statistically analyzed in B. Mean ± SEM, n = 6, *p < 0.05 (Student’s t-test). (C, D) Cell lysates from BV2 cells were immunoblotted demonstrating SB203580 decreased the levels of NLRP3, ASC, cleaved CASP1 and shown in D. Mean ± SEM, n = 6, *p < 0.05. (E, F) Levels of caspase-1 and IL-1β in tissue homogenates of wild type, α-synuclein A53T-tg and α-synuclein A53T-tg treated with SB203580 were assessed by ELISA assay. (G) The level of mRNA was detected by qPCR. (H) The mouse NLRP3 contains two noncanonical KFERQ-like pentapeptide (355LEKLQ359, 603QIRLE607, 795QKLVE799 and 989EVLKQ993). (I-K) Cell lysates from primary microglia were immunoblotted to detect the LAMP2A and NLRP3 after starvation and analyzed in J and K. Mean ± SEM, n = 3, *p < 0.05. (L, M) Cell lysates from primary microglia were immunoblotted to detect the levels of LAMP2A and NLRP3 after treatment with AR7 and QX77 and statistically analyzed in M. Mean ± SEM, n = 3, *p < 0.05.

**Additional file 3: Fig S3**. (A, B) Lysates from SNpc of 9 months α-synuclein A53T-tg or wild-type mice were subjected to subcellular fractionation, the nuclear and cytosolic fractions were immunoblotted using the indicated antibodies to determine the levels of TFEB. Data are shown in C. Mean ± SEM, n = 3. *p < 0.05. (C, D) BV2 cells were labeled with Lysosome tracker and visualized lysosome biogenesis under a microscope demonstrating SB203580 increased the levels of lysosome biogenesis and shown in E. Mean ± SEM, n = 10, *p < 0.05. (E) EM images of the lysosomal morphology were shown after SB203580 treatment. (F) The autophagy-related proteins LC3 and P62 were detected in SNpc of brain tissue. (G)Levels of IL-1β and IL-18 were assessed by ELISA. Data were performed using the Student’s unpaired t-test. (H, I) Cell lysates from BV2 cells were immunoblotted to detect the levels of pSer211TFEB and statistically analyzed in M. Mean ± SEM, n = 3.

**Additional file 4: Fig S4**. (A, B) BV2 cells were labeled with Lysosome tracker and visualized lysosome biogenesis under a microscope demonstrating si-p38 increased the levels of lysosome biogenesis and shown in B. Mean ± SEM, n = 10, *p < 0.05. (C, D) Cell lysates from BV2 cells were immunoblotted demonstrating si-p38 decreased the levels of NLRP3, cleaved CASP1 and shown in D. Mean ± SEM, n = 6, *p < 0.05. (E, F) Cell lysates from BV2 cells were immunoblotted demonstrating TFEB decreased the levels of NLRP3, cleaved CASP1 and shown in F. Mean ± SEM, n = 6, *p < 0.05. (G, H) Cell lysates from BV2 cells were immunoblotted and shown in H. Mean ± SEM, n = 6, *p < 0.05. (I, J) Levels of IL-1β and IL-18 in conditional mediate of BV2were assessed by ELISA assay. Data were performed in F. (K, L) Cell lysates from BV2 cells were immunoblotted demonstrating autophagy inhibitor CQ eliminate the effect of decreased NLRP3 from SB20350 and shown in L. Mean ± SEM, n = 6, *p < 0.05.

**Additional file 5: Fig S5.** (A, B) Cell apoptosis of SN 4741 cells was detected by flow cytometry dyeing with Annexin V-FITC/PI, followed by the treatment with conditioned medium from BV2. Mean ± SEM, n = 3, *p < 0.05. (C) Balance beam foot slips were quantified after PBS or MCC950 injection demonstrating the protective effect of MCC950. (D) Results of the pole test were quantified after PBS or MCC950 injection demonstrating the protective effect of MCC950. (E, F) Immunofluorescence (IF) staining of synapsin-1 (SYN-1) in the SNpc of mice. Mean ± SEM, n = 6, *p < 0.05.

## Data Availability

All data in this study are included in this published article and its supplementary information files.

## Abbreviations

PD: Parkinson’s disease
SNpc: Substantia nigra pars compacta
TFEB: Transcription factor EB
CMA: Chaperone-mediated autophagy
MAPKs: Mitogen-activated protein kinases
TLR: Toll-like receptor
Aβ: Beta-amyloid
CASP1: Caspase1
AD: Alzheimer’s disease
ALP: Autophagy-lysosomal pathway
