# Supplementary figures and images for: p38-TFEB pathways promote microglia activation through inhibiting CMA-mediated NLRP3 degradation in Parkinson's disease
Source: J Neuroinflammation. 2021 Dec 20;18:295. doi: 10.1186/s12974-021-02349-y (PMC8686293; doi:10.1186/s12974-021-02349-y)

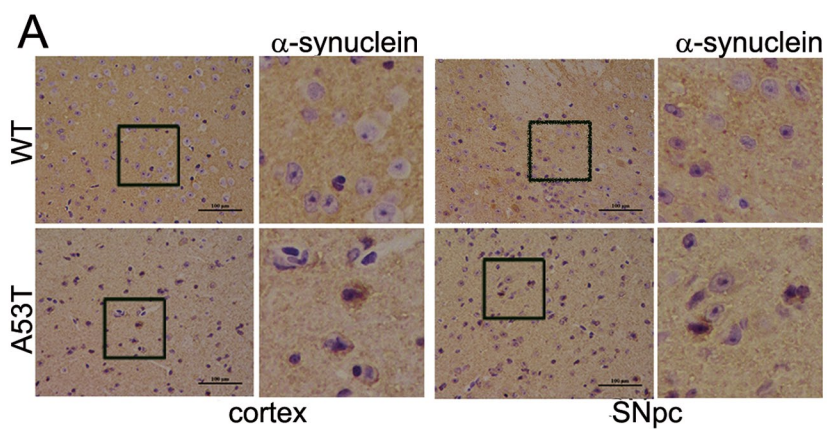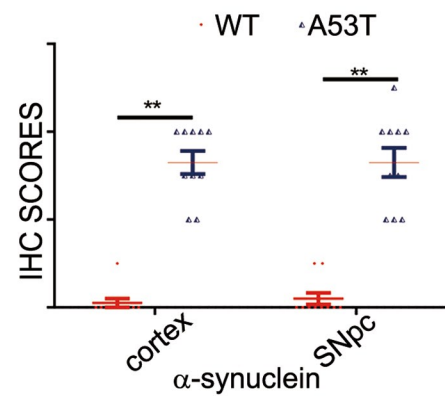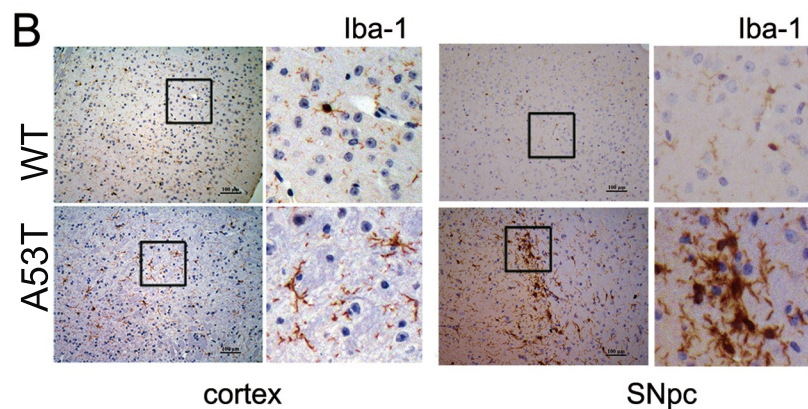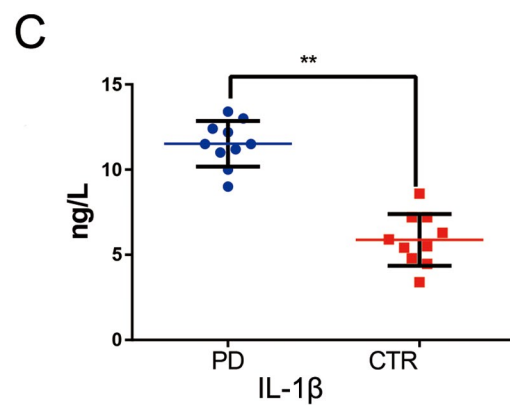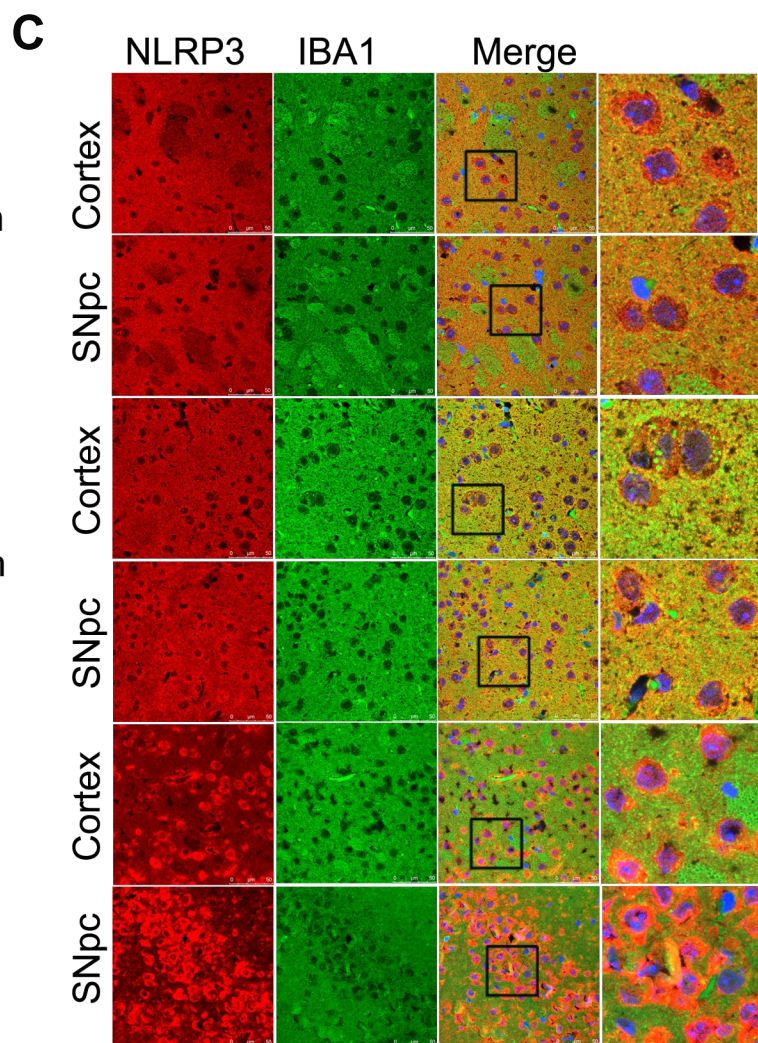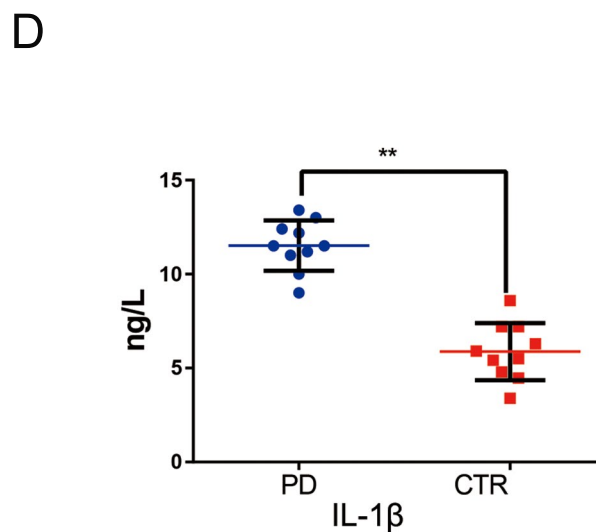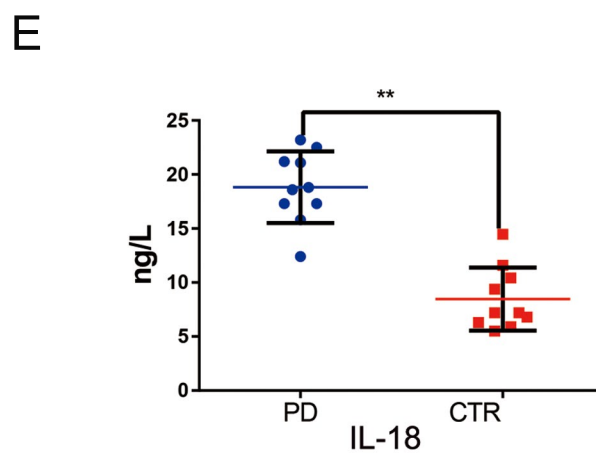

Supplement: Supplementary file 1 — Additional file 1: Fig S1. (A) Immunohistochemistry (IHC) demonstrating increased α-synuclein in the cortex and SNpc of 9 months α-synuclein A53T-tg or wild-type mice. Statistical analysis of the average score of α-synuclein staining between α-synuclein A53T-tg and wild-type mice. *p<0.05 (Student’s t-test). (B) Immunohistochemistry (IHC) demonstrating increased IBA-1 in the cortex and SNpc of 9 months α-synuclein A53T-tg or wild-type mice. (C) The co-staining between Iba1 and NLRP3 was detected by IF, which the dynamic changes of NLRP3 inflammasome activation in microglia from 3 months to 9 months of age. (D, E) Levels of IL-1β and IL-18 in serum were assessed by ELISA assay in 10 patients suffering from PD and 10 control. Data were performed using the Student’s unpaired t-test. [file 12974_2021_2349_MOESM1_ESM.pdf]

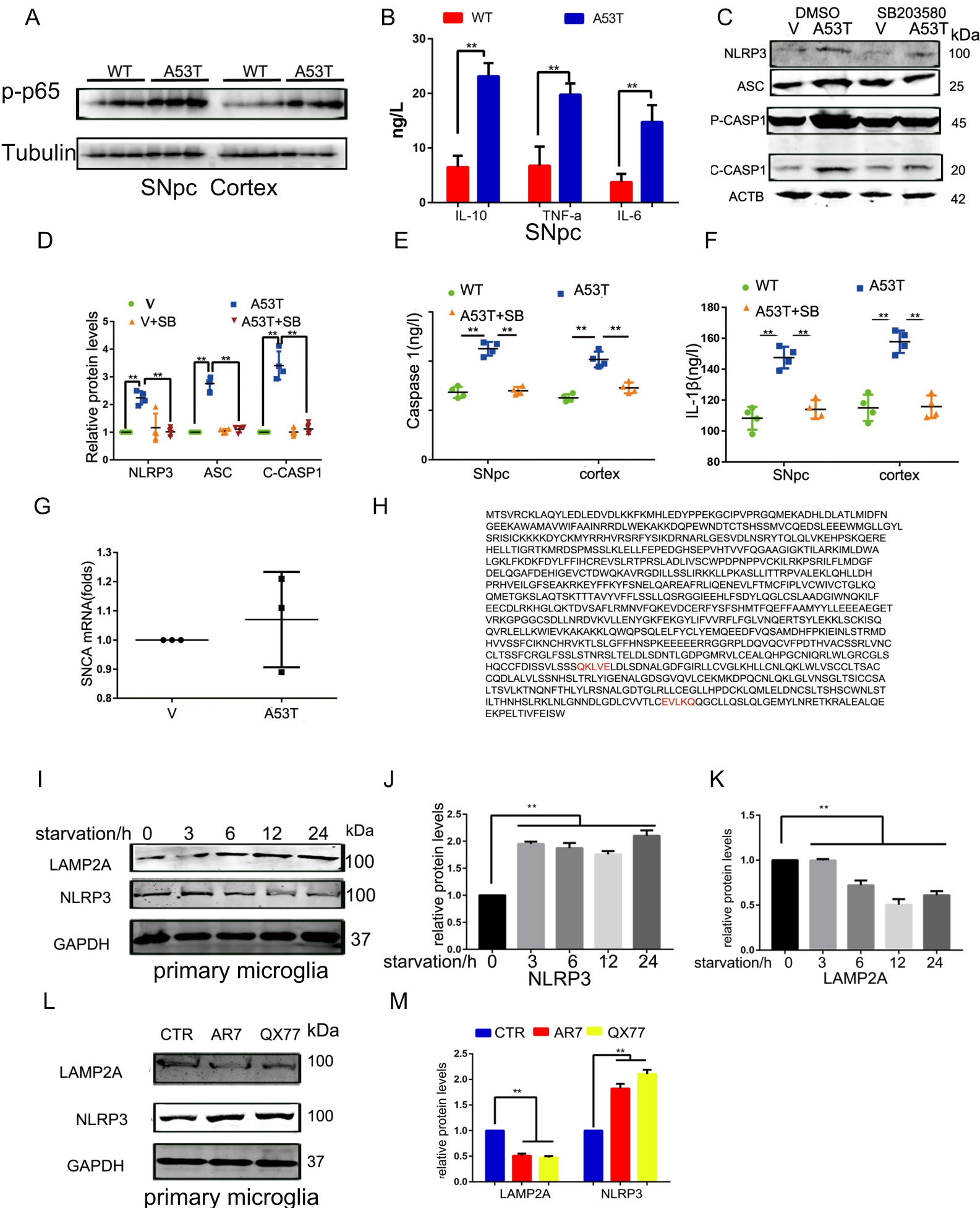

Supplement: Supplementary file 2 — Additional file 2: Fig S2 Lysates from the cortex and SNpc of mice were immunoblotted using the indicated antibodies. The protein levels of p-p65 were statistically analyzed in B. Mean ± SEM, n = 6, *p < 0.05 (Student’s t-test). (C, D) Cell lysates from BV2 cells were immunoblotted demonstrating SB203580 decreased the levels of NLRP3, ASC, cleaved CASP1 and shown in D. Mean ± SEM, n = 6, *p < 0.05. (E, F) Levels of caspase-1 and IL-1β in tissue homogenates of wild type, α-synuclein A53T-tg and α-synuclein A53T-tg treated with SB203580 were assessed by ELISA assay. (G) The level of mRNA was detected by qPCR. (H) The mouse NLRP3 contains two noncanonical KFERQ-like pentapeptide (355LEKLQ359, 603QIRLE607, 795QKLVE799 and 989EVLKQ993). (I-K) Cell lysates from primary microglia were immunoblotted to detect the LAMP2A and NLRP3 after starvation and analyzed in J and K. Mean ± SEM, n = 3, *p < 0.05. (L, M) Cell lysates from primary microglia were immunoblotted to detect the levels of LAMP2A and NLRP3 after treatment with AR7 and QX77 and statistically analyzed in M. Mean ± SEM, n = 3, *p < 0.05. [file 12974_2021_2349_MOESM2_ESM.pdf]

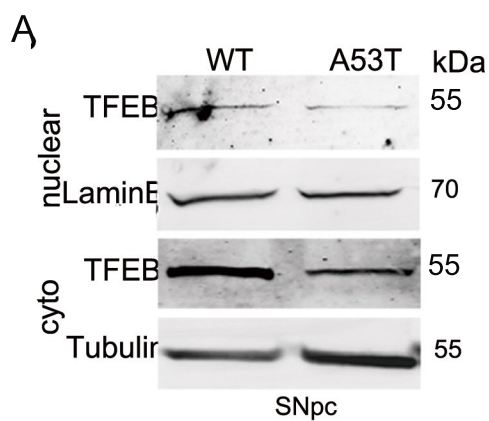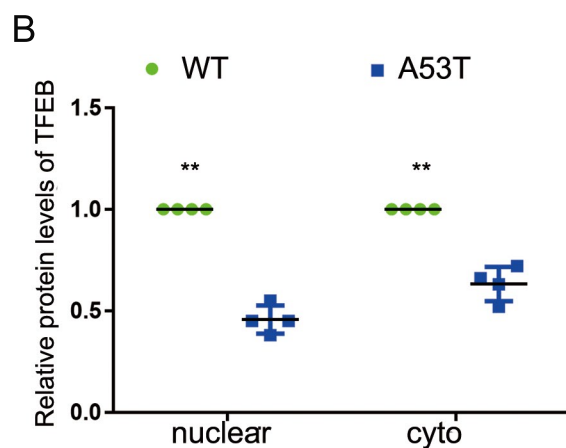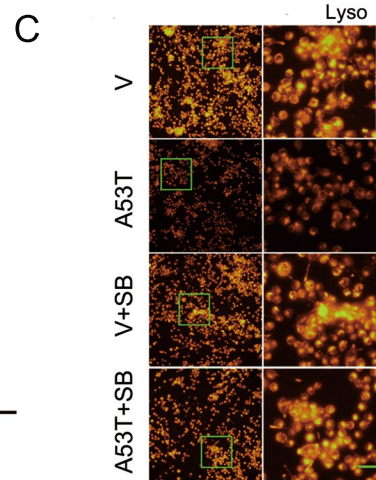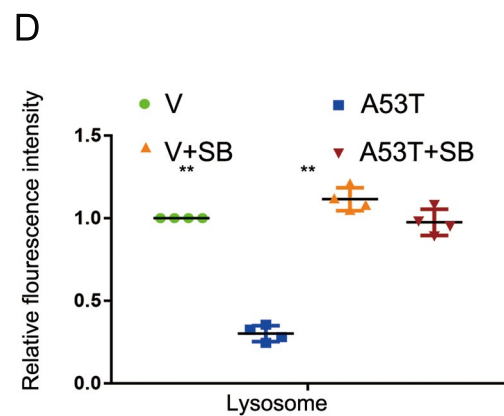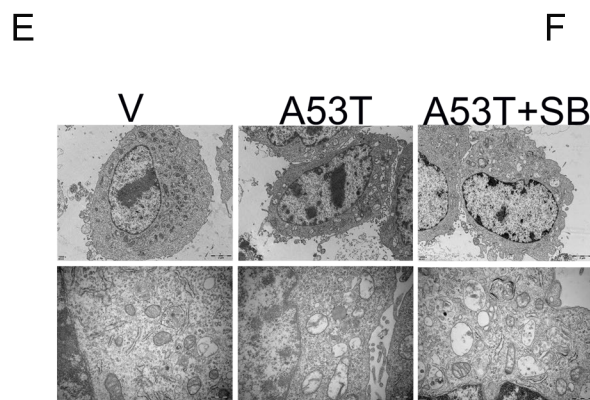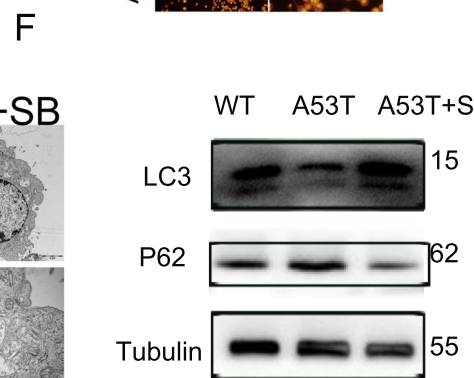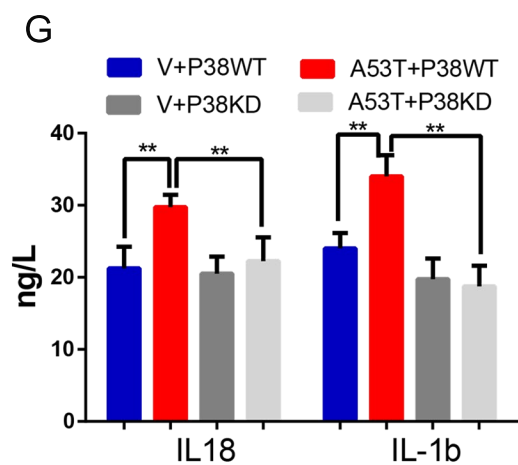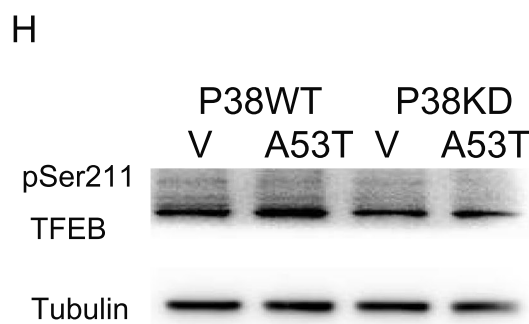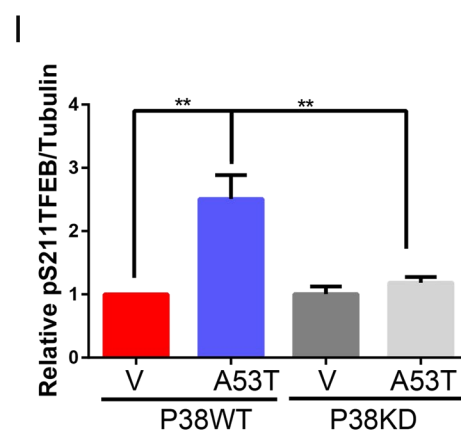

Supplement: Supplementary file 3 — Additional file 3: Fig S3. (A, B) Lysates from SNpc of 9 months α-synuclein A53T-tg or wild-type mice were subjected to subcellular fractionation, the nuclear and cytosolic fractions were immunoblotted using the indicated antibodies to determine the levels of TFEB. Data are shown in C. Mean ± SEM, n = 3. *p < 0.05. (C, D) BV2 cells were labeled with Lysosome tracker and visualized lysosome biogenesis under a microscope demonstrating SB203580 increased the levels of lysosome biogenesis and shown in E. Mean ± SEM, n = 10, *p < 0.05. (E) EM images of the lysosomal morphology were shown after SB203580 treatment. (F) The autophagy-related proteins LC3 and P62 were detected in SNpc of brain tissue. (G)Levels of IL-1β and IL-18 were assessed by ELISA. Data were performed using the Student’s unpaired t-test. (H, I) Cell lysates from BV2 cells were immunoblotted to detect the levels of pSer211TFEB and statistically analyzed in M. Mean ± SEM, n = 3. [file 12974_2021_2349_MOESM3_ESM.pdf]

**A**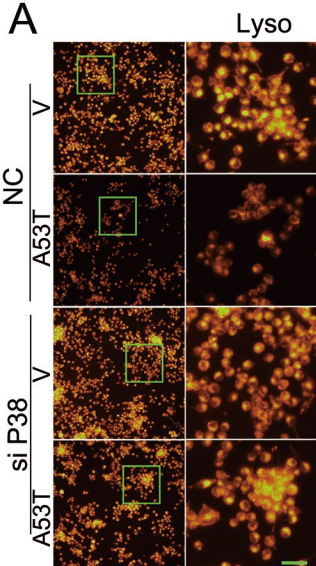**B**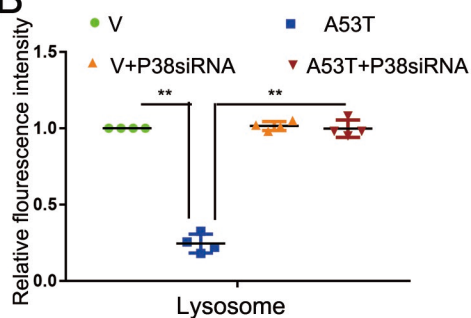**C**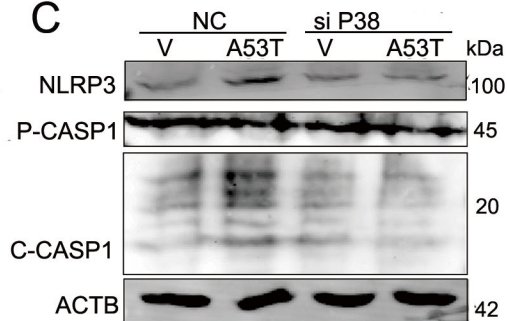**D**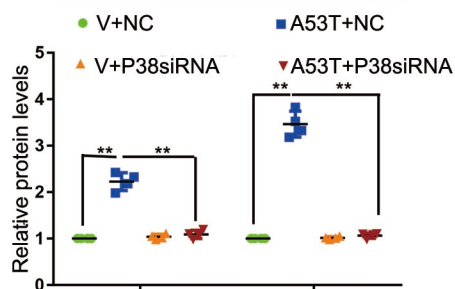**E**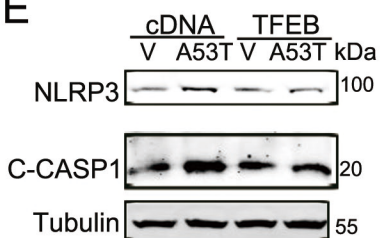**F**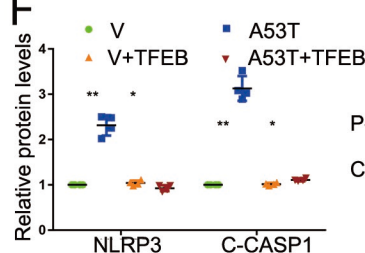**G**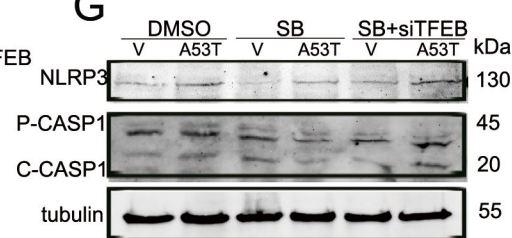**H**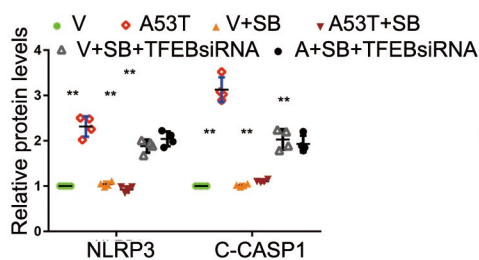**I**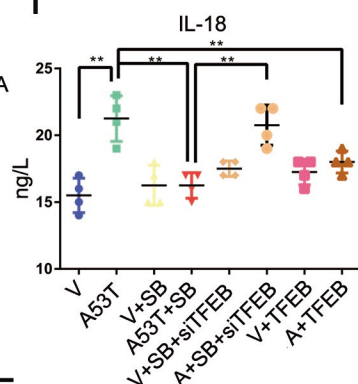**J**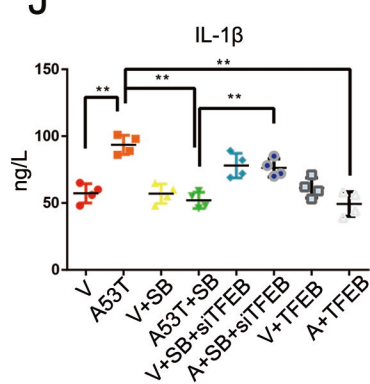**K**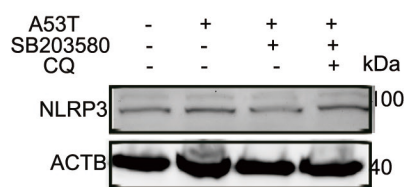**L**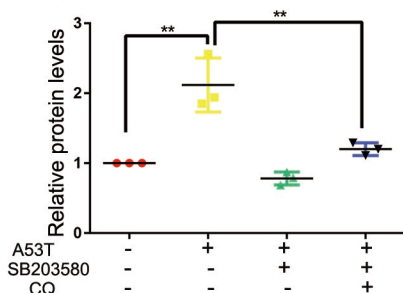

Supplement: Supplementary file 4 — Additional file 4: Fig S4. (A, B) BV2 cells were labeled with Lysosome tracker and visualized lysosome biogenesis under a microscope demonstrating si-p38 increased the levels of lysosome biogenesis and shown in B. Mean ± SEM, n = 10, *p < 0.05. (C, D) Cell lysates from BV2 cells were immunoblotted demonstrating si-p38 decreased the levels of NLRP3, cleaved CASP1 and shown in D. Mean ± SEM, n = 6, *p < 0.05. (E, F) Cell lysates from BV2 cells were immunoblotted demonstrating TFEB decreased the levels of NLRP3, cleaved CASP1 and shown in F. Mean ± SEM, n = 6, *p < 0.05. (G, H) Cell lysates from BV2 cells were immunoblotted and shown in H. Mean ± SEM, n = 6, *p < 0.05. (I, J) Levels of IL-1β and IL-18 in conditional mediate of BV2were assessed by ELISA assay. Data were performed in F. (K, L) Cell lysates from BV2 cells were immunoblotted demonstrating autophagy inhibitor CQ eliminate the effect of decreased NLRP3 from SB20350 and shown in L. Mean ± SEM, n = 6, *p < 0.05. [file 12974_2021_2349_MOESM4_ESM.pdf]

**A**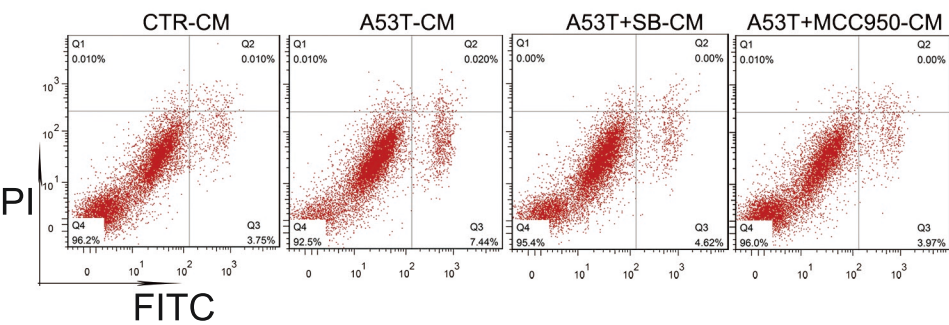**B**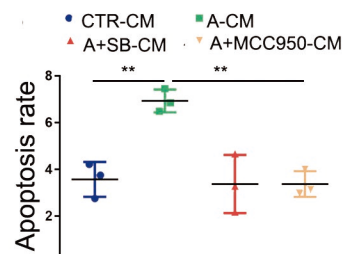**C**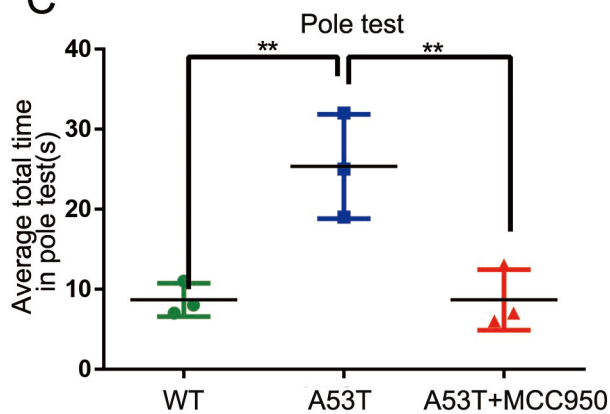**D**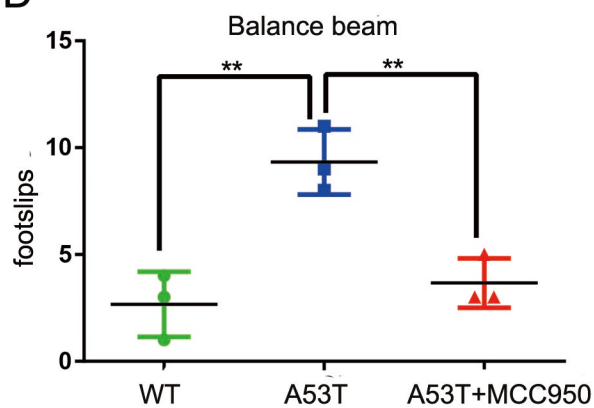**E**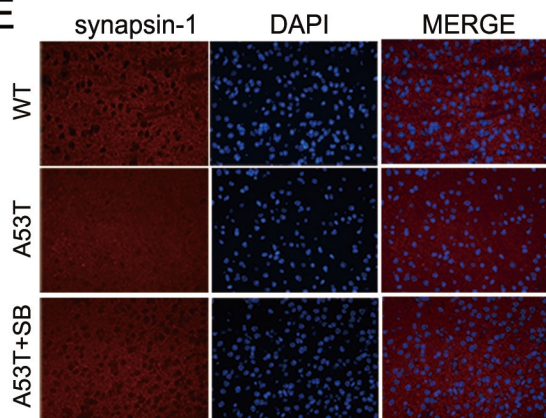**F**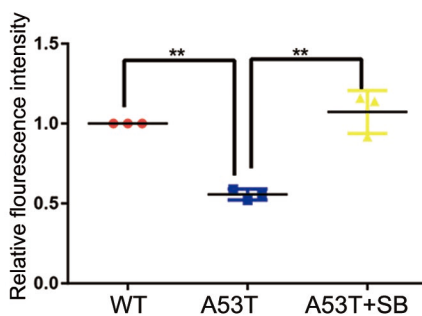

Supplement: Supplementary file 5 — Additional file 5: Fig S5. (A, B) Cell apoptosis of SN 4741 cells was detected by flow cytometry dyeing with Annexin V-FITC/PI, followed by the treatment with conditioned medium from BV2. Mean ± SEM, n = 3, *p < 0.05. (C) Balance beam foot slips were quantified after PBS or MCC950 injection demonstrating the protective effect of MCC950. (D) Results of the pole test were quantified after PBS or MCC950 injection demonstrating the protective effect of MCC950. (E, F) Immunofluorescence (IF) staining of synapsin-1 (SYN-1) in the SNpc of mice. Mean ± SEM, n = 6, *p < 0.05. [file 12974_2021_2349_MOESM5_ESM.pdf]
